# Preclinical evaluation of FAP-2286 for fibroblast activation protein targeted radionuclide imaging and therapy

**DOI:** 10.1007/s00259-022-05842-5

**Published:** 2022-05-24

**Authors:** Dirk Zboralski, Aileen Hoehne, Anne Bredenbeck, Anne Schumann, Minh Nguyen, Eberhard Schneider, Jan Ungewiss, Matthias Paschke, Christian Haase, Jan L. von Hacht, Tanya Kwan, Kevin K. Lin, Jan Lenore, Thomas C. Harding, Jim Xiao, Andrew D. Simmons, Ajay-Mohan Mohan, Nicola Beindorff, Ulrich Reineke, Christiane Smerling, Frank Osterkamp

**Affiliations:** 1 3B Pharmaceuticals GmbH, Magnusstraße 11, 12489 Berlin, Germany; 2 Clovis Oncology, Inc, Boulder, CO USA; 3 Berlin Experimental Radionuclide Imaging Center, Charité - Universitätsmedizin Berlin, Berlin, Germany

**Keywords:** FAP, CAF, PTRT, Theranostic

## Abstract

**Purpose:**

Fibroblast activation protein (FAP) is a membrane-bound protease that has limited expression in normal adult tissues but is highly expressed in the tumor microenvironment of many solid cancers. FAP-2286 is a FAP-binding peptide coupled to a radionuclide chelator that is currently being investigated in patients as an imaging and therapeutic agent. The potency, selectivity, and efficacy of FAP-2286 were evaluated in preclinical studies.

**Methods:**

FAP expression analysis was performed by immunohistochemistry and autoradiography on primary human cancer specimens. FAP-2286 was assessed in biochemical and cellular assays and in *in vivo* imaging and efficacy studies, and was further evaluated against FAPI-46, a small molecule–based FAP-targeting agent.

**Results:**

Immunohistochemistry confirmed elevated levels of FAP expression in multiple tumor types including pancreatic, breast, and sarcoma, which correlated with FAP binding by FAP-2286 autoradiography. FAP-2286 and its metal complexes demonstrated high affinity to FAP recombinant protein and cell surface FAP expressed on fibroblasts. Biodistribution studies in mice showed rapid and persistent uptake of ^68^Ga-FAP-2286, ^111^In-FAP-2286, and ^177^Lu-FAP-2286 in FAP-positive tumors, with renal clearance and minimal uptake in normal tissues. ^177^Lu-FAP-2286 exhibited antitumor activity in FAP-expressing HEK293 tumors and sarcoma patient-derived xenografts, with no significant weight loss. In addition, FAP-2286 maintained longer tumor retention and suppression in comparison to FAPI-46.

**Conclusion:**

In preclinical models, radiolabeled FAP-2286 demonstrated high tumor uptake and retention, as well as potent efficacy in FAP-positive tumors. These results support clinical development of ^68^Ga-FAP-2286 for imaging and ^177^Lu-FAP-2286 for therapeutic use in a broad spectrum of FAP-positive tumors.

**Supplementary Information:**

The online version contains supplementary material available at 10.1007/s00259-022-05842-5.

## Introduction

Fibroblast activation protein (FAP) is a single-pass type II transmembrane glycoprotein (1), with a large extracellular domain composed of an α/β-hydrolase and an 8-bladed β-propeller (2). As a member of the S9 prolyl oligopeptidase family, FAP has post-proline exopeptidase activity similar to dipeptidyl peptidase 4 (DPP4), and an additional endopeptidase activity like prolyl endopeptidase (PREP) (3,4).

FAP is expressed during embryonic development, with highly restricted expression in healthy adult tissues (5,6). However, FAP upregulation can occur in diseases associated with activated stroma including wound healing, rheumatoid arthritis, cirrhosis, pulmonary fibrosis, and solid cancers (7–10). In most epithelial cancers, FAP is selectively expressed on the cell surface of cancer-associated fibroblasts (CAFs) present in the tumor microenvironment (11). These CAFs play important roles in tumor growth and metastasis through their effects on angiogenesis, the extracellular matrix, and the immune system (12). In addition, in several tumors of mesenchymal origin, notably sarcoma and mesothelioma, FAP expression has also been observed on the neoplastic cells themselves (11) and is similarly associated with promoting tumor progression and metastasis (6). Given its restricted expression profile and functions, FAP is a promising target for the selective delivery of anticancer therapies to tumors of a broad range of cancer indications (13–16).

Many FAP-targeting agents have been developed including small molecule inhibitors and immunoglobulins. Of the antibody-based therapies, sibrotuzumab has been evaluated in a clinical trial and demonstrated no objective clinical responses, while the antibody drug conjugates FAP5-DM1 and OMTX705 have yet to be investigated in patients (16,17). In addition, the radioimmunoconjugate ESC11 designed for diagnostic and therapeutic applications has shown efficacy in a preclinical melanoma xenograft model (15). However, antibodies are well known to have an extended blood circulating half-life which may elicit greater normal tissue toxicity, particularly in the bone marrow when conjugated to a radionuclide. In contrast, small molecular weight radioconjugates have the advantage of rapid tumor delivery coupled to fast systemic clearance to potentially circumvent this toxicity.

One approach to targeting FAP is through radiolabeled theranostic agents, integrating both noninvasive disease diagnosis and treatment. Validated targeted radiopharmaceuticals include LUTATHERA® (^177^Lu-dotatate; Advanced Accelerator Applications), a cyclic peptide targeting somatostatin subtype 2 receptor (SSTR2) armed with the β-emitting radionuclide lutetium-177 (^177^Lu), which has been approved for treatment of SSTR2-positive patients with gastroenteropancreatic neuroendocrine tumors (GEP-NET) (18); and ^177^Lu-PSMA-617, a small molecule targeting prostate-specific membrane antigen (PSMA) linked to ^177^Lu, which recently met both primary survival endpoints in the phase III VISION trial for PSMA-positive metastatic castration-resistant prostate cancer (mCRPC) (19).

FAP is an emergent target in the radiopharmaceutical field and has been referred to as “the next billion dollar nuclear theranostics target” (20). This description originates from the development of the FAP inhibitor (FAPI) series of small molecule quinoline-based radiotracers (21) that have shown high specificity across various primary and metastatic tumors (22,23). The diagnostic utility of ^68^Ga-FAPI–based positron emission tomography (PET) has been established in various cancer types, demonstrating substantial accumulation in cancer of unknown primary (CUP), sarcoma, esophageal cancer, breast cancer, and cholangiocarcinoma (22). In addition, ^68^Ga-FAPI-04 appears to outperform ^18^F-fluorodeoxyglucose (^18^F-FDG) in discriminating the primary and distant metastatic lesions in certain indications (24–26).

Further structural modifications of the FAPI series of compounds are currently ongoing to improve pharmacology and allow the attachment of alternative radionuclides (27,28). In parallel, multiple academic and commercial entities have recently described highly related FAP-targeting molecules with similar results (29,30), including the lead therapeutic compound FAPI-46 with a methylamino alteration in the linker region. However, the FAPI radiotracers are established around UAMC1110 (31), a small molecule inhibitor of FAP, and exhibit fast clearance from tumors, limiting their potential therapeutic effectiveness.

To address the low tumor retention of the FAPI series, we developed the FAP-2286 compound, based on a novel class of FAP-targeting modalities, that utilizes cyclic peptides as binding motifs. Cyclic peptides are known to have potentially improved biological properties over linear counterparts, including greater binding affinity and selectivity due to their conformational rigidity and increased plasma stability. FAP-2286 is composed of such a cyclic peptide, which is linked to a tetraazacyclododecane tetraacetic acid (DOTA) allowing chelation of radionuclides for imaging or therapeutic applications. For imaging, the radionuclides gallium-68 (^68^Ga) or indium-111 (^111^In) were chelated to FAP-2286 for use as a PET or single-photon emission computed tomography (SPECT) imaging agent, respectively. For therapeutic use, ^177^Lu was chelated to FAP-2286. Herein, the *in vitro* and *in vivo* characterization of FAP-2286 and its metal complexes are reported and compared to FAPI-46.

## Material and methods

Detailed information on materials and methods including peptide synthesis are described in the Supplementary Materials and Methods.

### Immunohistochemistry

Formalin-fixed paraffin-embedded (FFPE) tissue microarrays (US Biomax) and whole sections (Indivumed, Tissue Solutions, and Discovery Life Sciences) were stained with FAP antibody (SP325, Abcam; 1:50) according to the manufacturer recommendation. Staining was performed on the Bond Rx Autostainer (Leica Biosystems) and detection by the Bond Polymer Refine Detection (Leica Biosystems) according to manufacturer’s protocol. Whole slide scanning was performed on the Aperio AT2 (Leica Biosystems), and analyses were conducted using Aperio ImageScope (Leica Biosystems), Visiopharm Integrator System (Visiopharm), or HALO (Indica Labs) image analysis platform.

### Autoradiography

*In vitro* FAP autoradiography was performed as previously described with minor modifications (32). The 20-µm-thick frozen tissue sections were incubated with ^111^In-labeled FAP-2286 (15 MBq/nmol). Subsequently, an x-ray film (Biomax MR films; Carestream Health) was placed onto positioned slides and exposed for 2 days. Relative optical film density was determined using MCID™ software (InterFocus) and correlated with known amounts of radioactivity via a separately recorded calibration curve.

### *In vitro* assays

#### Surface plasmon resonance assay (SPR)

The binding kinetics of FAP-2286 to antibody-immobilized human FAP (Sino Biological) or mouse FAP (R&D Systems) was measured by SPR and calculated using single-cycle kinetic measurements.

#### Cell-based binding assay

FAP-expressing WI-38 fibroblasts (ECACC) were co-incubated for 1 h at 4 °C with Cy5-labeled FAP-binding competitor peptide and various concentrations of FAP-2286. Median fluorescence intensity was measured by flow cytometry on Attune NxT (Thermo Fisher Scientific), and 4PL curve fitting and half-maximal inhibitory concentration (IC_50_) calculations were performed using the ActivityBase software (IDBS).

#### Protease activity assay

Recombinant human and mouse FAP (R&D Systems) were incubated with various concentrations of FAP-2286 before a fluorophore-labeled specific substrate (Eurogentec; (33)) was added to the well, and fluorescence output was measured for 5 min at 37 °C on SpectraMax M5 (Molecular Devices).

#### Plasma stability assay

FAP-2286 was incubated in human or mouse plasma at 37 °C for 24 h. Compound concentration was measured using HPLC–MS/MS (Agilent Technologies) and percent concentration remaining was calculated.

### Murine tumor models

HEK-FAP cell line was generated using the vector pTAR to insert FAP complementary DNA by recombinase-mediated cassette exchange into the pre-tagged HEK293 cells (InSCREENeX). For *in vivo* HEK-FAP studies, female nude or SCID beige mice (Charles River Laboratories) were subcutaneously implanted with 5 × 10^6^ HEK-FAP cells in 150 µL serum-free media:Matrigel (1:1) (Corning). For the patient-derived xenograft (PDX) efficacy study, female NMRI nu/nu mice (Janvier Labs) were subcutaneously implanted with Sarc4809 fragments (Experimental Pharmacology & Oncology Berlin-Buch). All animal experiments were carried out under licenses approved by the respective authorities and in compliance with guidelines on animal welfare.

### *In vivo* biodistribution

Thirty megabecquerels (30 MBq/nmol) ^111^In-FAP-2286 (*n* = 9) or ^111^In-FAPI-04 (*n* = 6) was injected intravenously (IV) into HEK-FAP tumor-bearing mice and the distribution of the radiotracers was assessed using the NanoSPECT/computed tomography (CT) system (Mediso). For PET imaging, 10 MBq of ^68^Ga-FAP-2286 or ^68^Ga-FAPI-46 (10 MBq/nmol) was given IV into HEK-FAP tumor-bearing mice (*n* = 3) followed by PET/CT analysis using the nanoScan PET/CT system (Mediso). For SPECT imaging of ^177^Lu-labeled FAP-2286 or ^177^Lu-labeled FAPI-46, 30 MBq was administered IV (30 MBq/nmol; *n* = 3) and the distribution of the radiotracers was assessed using the nanoScan SPECT/CT system (Mediso).

### Efficacy studies

A single dose of the indicated treatments was administered IV on day 0, with vehicle (saline), nonradioactive ^nat^Lu-FAP-2286, 30 or 60 MBq ^177^Lu-FAP-2286 (30 MBq/nmol and 60 MBq/nmol, respectively), or 30 MBq ^177^Lu-FAPI-46 (30 MBq/nmol). Tumor length and width were measured by caliper, and tumor volume was calculated as 0.5 × (length × width^2^). Tumor growth and body weight were monitored 3 times weekly. Sarc4809 tumor growth was normalized to the mean tumor volume (MTV) at the time of injection for each group due to variability in tumor volume between the groups. Statistical analysis was performed using GraphPad Prism 8.4.

### Cellular internalization assay

FAP-2286 was labeled with Alexa Fluor 488 (AF488) at the C-terminus of the peptide. For FAPI-46, a free carboxy moiety of the DOTA was used for labeling using AF488 Cadaverine (Thermo Fisher Scientific).

#### Flow cytometry

HEK-FAP cells were incubated with various concentrations of AF488-labeled FAP-2286 or FAPI-46 for 1 h at 37 °C. Median fluorescence intensity (MFI) was measured on Attune NxT (Thermo Fisher Scientific).

#### Fluorescence microscopy

HEK-FAP cells were seeded on chamber slides (ibidi) and incubated with 5 nM of AF488-labeled FAP-2286 or FAPI-46 for 1 h at 37 °C. Cells were then washed and further incubated for various timepoints. Cell nuclei were stained with 1 µg/mL Hoechst 33342 dye. Lysosomes were stained using LysoTracker Deep Red (Thermo Fisher Scientific). Images were acquired by using the BZ X800E fluorescence microscope (Keyence) at 40 × magnification.

## Results

### FAP expression in human cancers

**Fig. 1:**
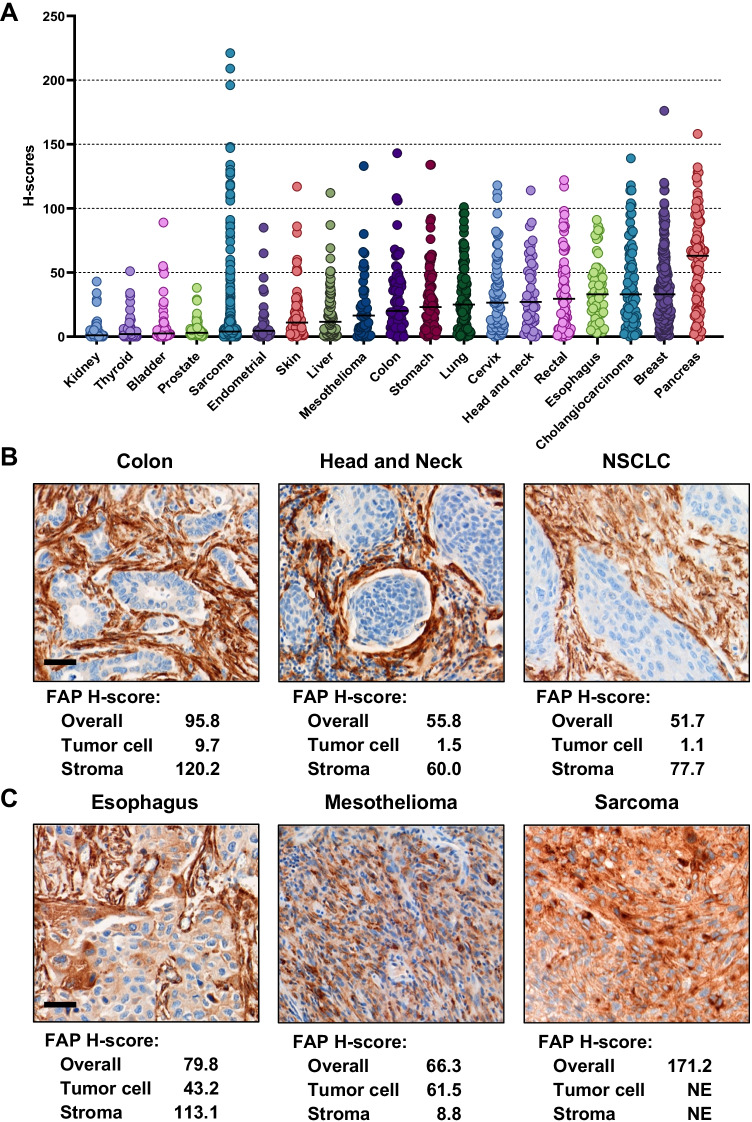
FAP expression in human solid cancers. *H*-score quantification of FAP expression in TMA is plotted in dot plot graph by cancer types (overall; ImageScope). Median values are indicated by horizontal black bars (**A**). Representative images of FAP expression limited to the stroma. *H*-score quantification of FAP expression in the whole tumor area (overall; Visiopharm) and in the tumor cell and stroma compartment (HALO). Scale bar, 50 µm (**B**). Representative images of FAP expression in tumor cells. *H*-score quantification of FAP expression in the whole tumor area (overall; Visiopharm) and in the tumor cell and stroma compartment (HALO). NE, not evaluable; NSCLC, non-small cell lung cancer. Scale bar, 50 µm (**C**)

Gene expression analysis across multiple cancer types using The Cancer Genome Atlas dataset (34) showed elevated FAP mRNA levels in different tumor types with the highest median observed in breast (1014 RNA-Seq by Expectation Maximization [RSEM], *n* = 1082) and pancreatic tumors (1213 RSEM, *n* = 177; Supplementary Fig. S1). FAP levels were validated by immunohistochemistry in human tumor samples on tissue microarrays (TMA) of 19 cancers (*n* = 1829 cores). Cell-surface staining patterns of varying intensity were detected in several indications with the highest median levels in pancreatic cancer (*H*-score = 63; *n* = 91) and the second highest in breast cancer (*H*-score = 33; *n* = 265), cholangiocarcinoma (*H*-score = 33; *n* = 83), and esophageal cancer (*H*-score = 33; *n* = 69; Fig. 1A).

For a more comprehensive examination, FAP immunohistochemistry was performed on whole FFPE tissue sections of 16 tumor types that included primary and metastatic samples of both low- and high-grade histology (*n* = 359, Supplementary Fig. S2A). High FAP expression (*H*-score cut-off of ≥ 30) was observed in multiple tumor types. More than 40% of pancreatic, cancer of unknown primary (CUP), salivary gland and mesothelioma tumors highly express FAP. High FAP expression was detected in both primary and metastatic samples (Supplementary Fig. S2B) and was independent of tumor grade (Supplementary Fig. S2C).

In addition, FAP levels in the tumor versus the stroma compartments were examined using automated imaging in a subset of cases with very high FAP staining across cancer types. This quantitative analysis confirmed that in most epithelial cancers, FAP expression was primarily confined to the CAFs in stroma (Fig. 1B). However, tumor cell staining was common in sarcoma and mesothelioma, and was also occasionally observed in epithelial tumors including HNSCC, CUP, esophageal and others (Fig. 1C).

### *In vitro* characterization of FAP-2286

**Fig. 2:**
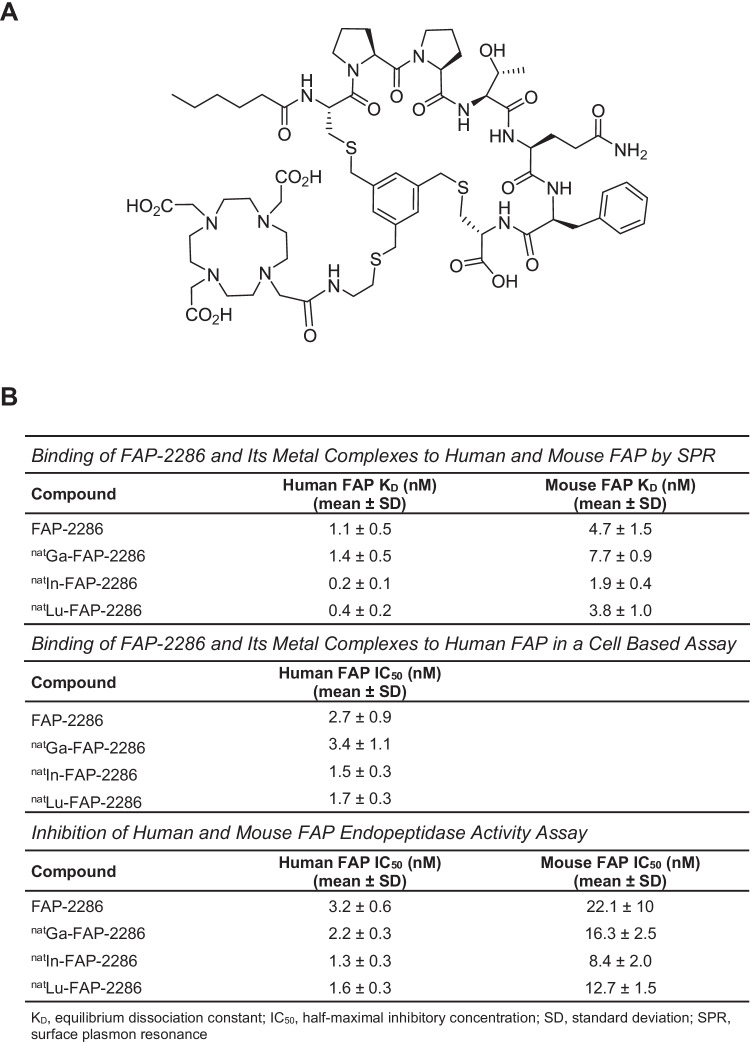
FAP-2286 structure (**A**) and *in vitro* characterization of FAP-2286 and its metal complexes summary table (**B**)

FAP-2286 is a FAP-binding cyclic peptide consisting of seven amino acids, wherein two cysteine residues are cyclized by an aromatic moiety linked to a DOTA chelator (Fig. 2A). The chelator can be labeled with radionuclides for imaging and therapeutic applications. The affinity and selectivity of FAP-2286 along with its complexes of natural nonradioactive metals (^nat^Ga-FAP-2286, ^nat^Lu-FAP-2286, and ^nat^In-FAP-2286) as surrogates of the three radiotracers were evaluated *in vitro*.

The binding of FAP-2286 and its natural metal complexes to recombinant human and mouse FAP was assessed by SPR (Fig. 2B). FAP-2286 demonstrated potent binding to human and mouse FAP, with mean equilibrium dissociation constant (*K*_*D*_) values of 1.1 and 4.7 nM, respectively. The high affinity was maintained for the metal complexes with *K*_*D*_ values ranging from 0.2 to 1.4 nM for human FAP and from 1.9 to 7.7 nM for mouse FAP. These results are consistent with the 89% amino acid sequence identity between the 2 species (5).

The binding of FAP-2286 to cell surface FAP was evaluated in a competition assay against a fluorophore-labeled competitor peptide using the human WI-38 fibroblast-like fetal lung cell line that endogenously expresses FAP. FAP-2286 binding to FAP markedly reduced fluorophore-labeled competitor peptide bound to cells with a mean IC_50_ of 2.7 nM, as measured by flow cytometry. The three metal complexes were comparably potent in inhibiting binding of the competitor peptide, with mean IC_50_ values ranging from 1.7 to 3.4 nM (Fig. 2B).

To further characterize binding *in vitro*, the potency of FAP-2286 was measured against FAP endopeptidase enzymatic activity on a fluorophore-labeled substrate. FAP-2286 inhibited FAP enzymatic activity with mean IC_50_ values of 3.2 and 22.1 nM against human and mouse FAP, respectively. Similarly, the mean IC_50_ values for the metal complexes ranged from 1.3 to 2.2 nM against human FAP and from 8.4 to 16.3 nM against mouse FAP (Fig. 2B).

Selectivity of FAP-2286 binding against the related family members DPP4 and PREP (4) was evaluated in enzymatic inhibition assays employing recombinant human proteins. FAP-2286 and its metal complexes showed minimal activity against the DPP4 and PREP proteases with IC_50_ values of  >1 and 10 μM for human recombinant PREP and DPP4, respectively, confirming lack of binding to related family homologs (Supplementary Table S1).

Confirmation of FAP-2286 peptide stability in plasma was also performed to assess potential degradation or metabolism of the radiotracers in the *in vivo* studies. Plasma stability assays revealed that FAP-2286 was stable for 24 h at 37 °C with > 95% and 85% retained in human and mouse plasma, respectively (Supplementary Fig. S3).

Taken altogether, the *in vitro* biochemical and cellular assays indicate that FAP-2286 and its metal complexes demonstrate potent and selective binding to FAP.

### *In vitro* autoradiography and *in vivo* imaging utilizing ^111^In-FAP-2286

**Fig. 3:**
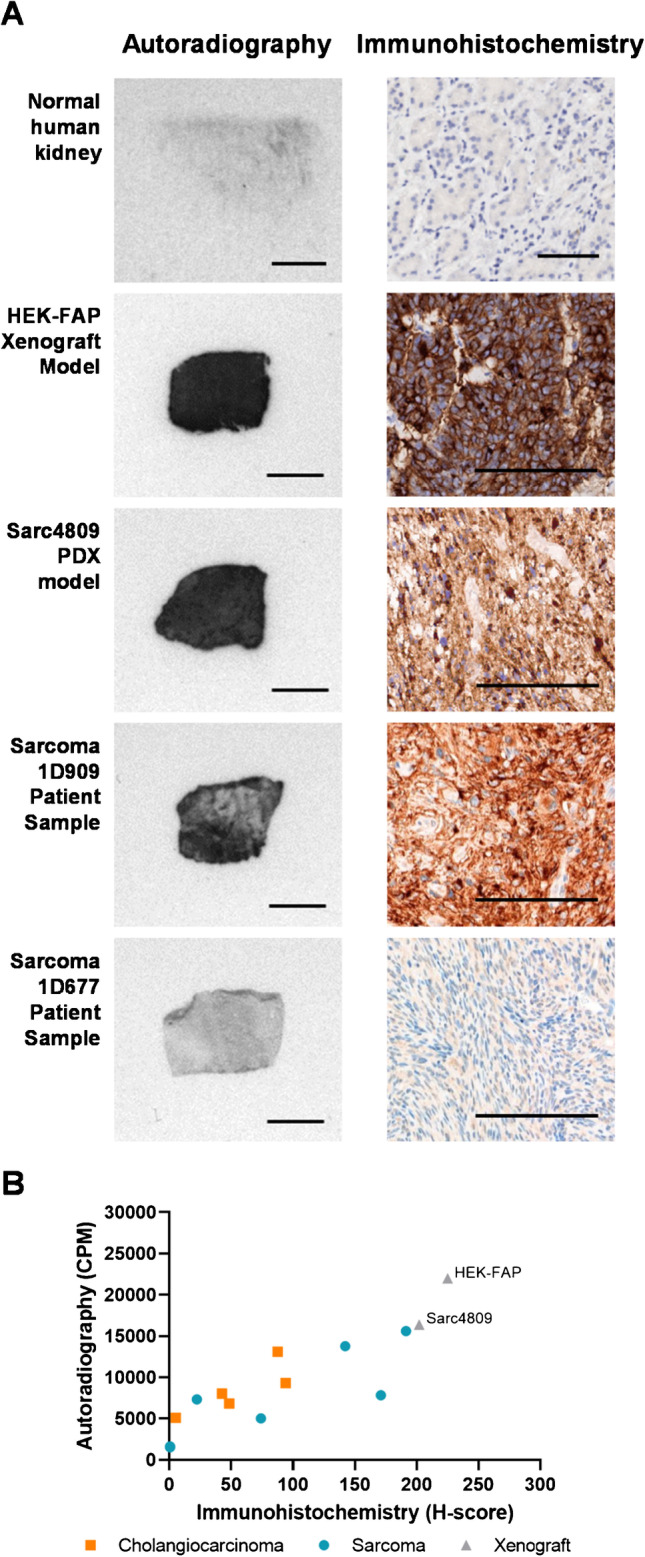
Correlation of FAP expression analysis by immunohistochemistry and autoradiography using ^111^In-FAP-2286. Representative images of patient and mouse xenograft tumors are shown by autoradiography (left, 500 µm) and immunohistochemistry (right, 100 μm) (**A**). FAP levels by autoradiography demonstrate correlation to immunohistochemistry in patient cholangiocarcinoma and sarcoma tumor sections (Pearson correlation coefficient *r* = 0.79, *P* = 0.002) (**B**)

Prior to *in vivo* evaluation of FAP-2286, the HEK-FAP xenograft was assessed for FAP expression by immunohistochemistry and FAP-2286 binding by *in vitro* autoradiography. The HEK-FAP tumor displayed high, homogeneous FAP levels, with a strong ^111^In-FAP-2286 binding signal by autoradiography (approximately 22,000 counts per minute [CPM]), which was corroborated by the immunohistochemistry *H*-score of 225. As a negative control, normal human kidney was utilized, and non-specific background levels were detected (Fig. 3A). In addition, a comparison between the 2 techniques was performed using a set of cholangiocarcinoma and sarcoma whole tissue sections that were selected for varying FAP *H*-scores (Fig. 3B). A direct correlation was observed in these human specimens with the two methods (Pearson *r* = 0.79, *P* = 0.002, *n* = 12).

**Fig. 4:**
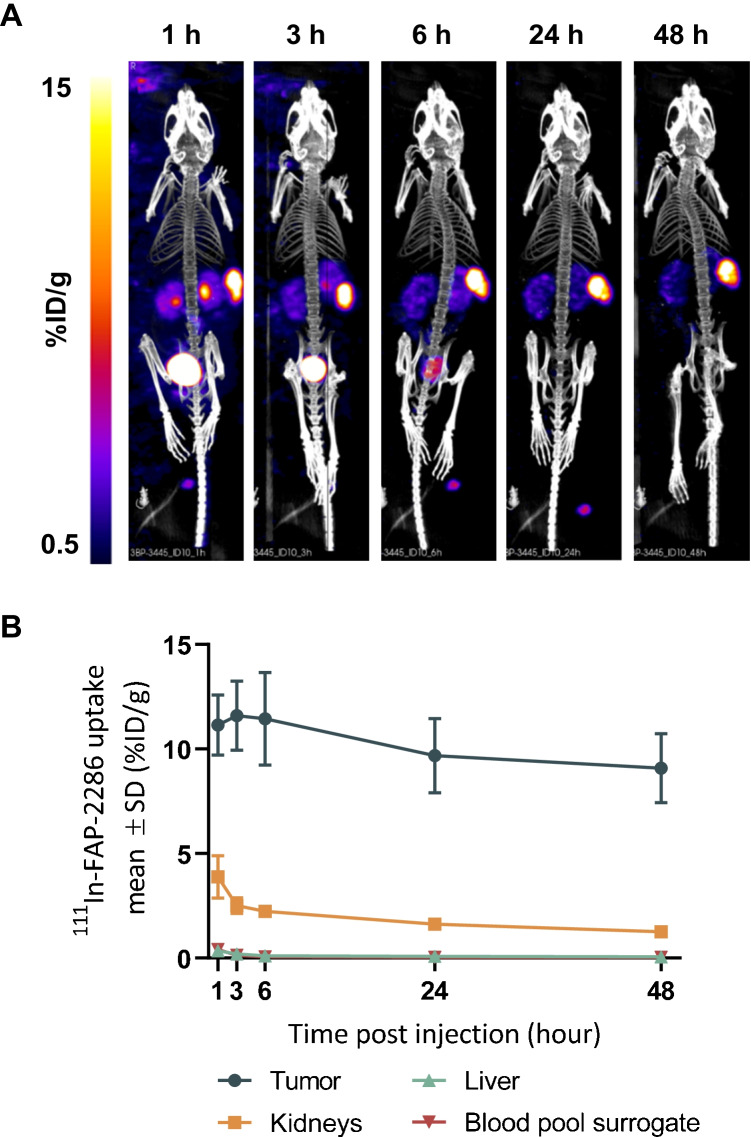
Imaging of HEK-FAP tumor-bearing mice with ^111^In-FAP-2286. SPECT images of one representative mouse at 5 different timepoints are shown (**A**). Quantification of ^111^In-FAP-2286 uptake as mean ± SD %ID/g (*n* = 9) in tumor, liver, kidney, and blood pool surrogate at various timepoints after injection (**B**)

The biodistribution of ^111^In-FAP-2286 was evaluated *in vivo* by SPECT imaging following a single dose of 30 MBq in HEK-FAP tumor bearing mice. The radiotracer was rapidly enriched within the HEK-FAP xenografts with low off-target accumulation and predominantly renal elimination (Fig. 4A). High tumor-to-background signal ratio was observed from 1 h post injection (p.i.) onward. At 1 h p.i. of ^111^In-FAP-2286, tumor uptake was 11.1 percent injected dose per gram (%ID/g). Accumulation of ^111^In-FAP-2286 was stably maintained in the tumors with 9.1%ID/g at 48 h p.i. (Fig. 4B, Supplementary Table S2). Normal tissues including liver, salivary gland, and shoulder showed very limited uptake. The organ with the highest non-target uptake was the kidney; however, an increasing tumor-to-kidney (T/K) ratio was observed over time with the highest differential uptake of 7.5 T/K obtained at 48 h p.i. (Supplementary Table S2).

### Efficacy of ^177^Lu-FAP-2286 in a HEK-FAP tumor model

**Fig. 5:**
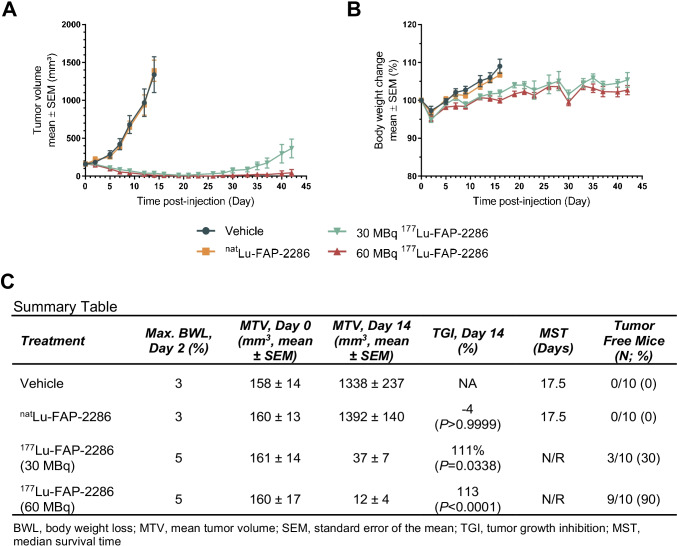
Efficacy of ^177^Lu-FAP-2286 treatment in the HEK-FAP tumor model. HEK-FAP tumor-bearing mice were treated with vehicle, ^nat^Lu-FAP-2286, or ^177^Lu-FAP-2286 at 30 or 60 MBq (*n* = 10 mice/group). Mean tumor volumes ± SEM (**A**), mean body weight change ± SEM (**B**), and a summary table (**C**) are shown

Based on the SPECT imaging observations, ^177^Lu-FAP-2286 antitumor efficacy was evaluated as a single dose of 30 or 60 MBq in HEK-FAP tumor bearing mice. Tumors in the control groups injected with vehicle or the natural nonradioactive metal-labeled ^nat^Lu-FAP-2286 reached a mean tumor volume (MTV) of 1338 and 1392 mm^3^, respectively, on day 14 p.i., when < 50% of the animals in the vehicle group were sacrificed due to tumor volumes reaching humane endpoint criteria. In contrast, the MTV for both ^177^Lu-FAP-2286 treatment groups were reduced to ≤ 37 mm^3^ on day 14 p.i. (Fig. 5A). Statistically significant antitumor activity was observed with tumor growth inhibition (TGI) of 111% and 113% (*P* < 0.05) in mice treated with 30 or 60 MBq ^177^Lu-FAP-2286, respectively, relative to the vehicle-treated group (Fig. 5C).

HEK-FAP xenografts were monitored for regrowth out to the end of the study (day 42), with 3 and 9 tumor-free (< 10 mm^3^) mice out of 10 in the 30 and 60 MBq ^177^Lu-FAP-2286 treatment groups, respectively. Furthermore, there was a statistically significant difference in the MTV on day 42 in animals treated with the low dose (365 mm^3^) versus the high dose (43 mm^3^) of ^177^Lu-FAP-2286, suggesting a dose response relationship (*P* < 0.01). The median survival time (MST) for the two ^177^Lu-FAP-2286 groups was not reached while the MST for the control groups was 17.5 days. The maximum body weight loss ranged from 3 to 5% on day 2 for all groups, with no ^177^Lu-FAP-2286–related weight loss observed (Fig. 5B).

### Efficacy of ^177^Lu-FAP-2286 in a sarcoma PDX model

The efficacy of ^177^Lu-FAP-2286 was evaluated in the Sarc4809 PDX model of sarcoma. Analogous to the HEK-FAP model, immunohistochemistry and autoradiography demonstrated high FAP expression in the Sarc4809 xenografts with a *H*-score of 202 and bound radioactivity of 16,000 CPM, respectively (Fig. 3).

**Fig. 6:**
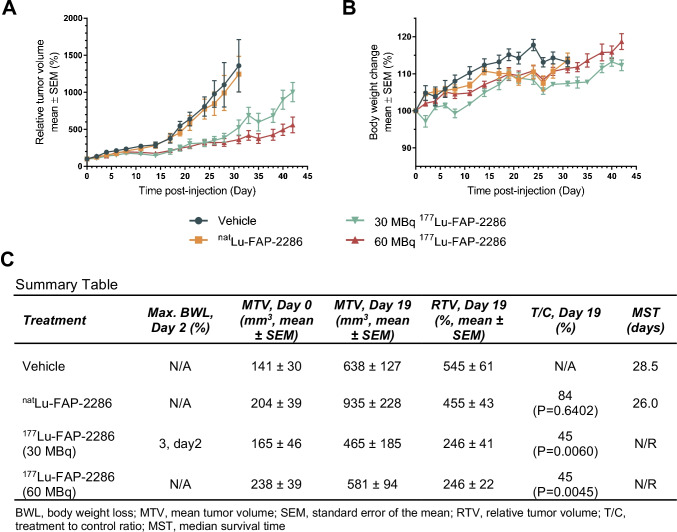
Efficacy of ^177^Lu-FAP-2286 treatment in the Sarc4809 tumor model. Sarc4809 tumor-bearing mice were treated with vehicle, ^nat^Lu-FAP-2286, or ^177^Lu-FAP-2286 at 30 or 60 MBq (*n* = 10 mice/group). Mean relative tumor volumes ± SEM (**A**), mean body weight change ± SEM (**B**), and a summary table (**C**) are shown

A single dose of vehicle, ^nat^Lu-FAP-2286, or ^177^Lu-FAP-2286 at 30 or 60 MBq was administered to Sarc4809 tumor bearing mice. Both dose levels of ^177^Lu-FAP-2286 resulted in significant antitumor activity (Fig. 6A). Because of starting tumor volume variability on day 0, all tumor volumes were normalized to the starting volumes to obtain relative tumor volume (RTV). On day 19 p.i., a single dose of 30 or 60 MBq ^177^Lu-FAP-2286 demonstrated significant tumor growth inhibition as the treatment-to-control ratio was 45% for both doses (*P* < 0.01). By day 42, there was a statistically significant difference in the RTV in animals treated with the low versus high dose of ^177^Lu-FAP-2286 (*P* = 0.018). The MST of vehicle and ^nat^Lu-FAP-2286 control groups was 28.5 and 26.0 days, respectively, but was not reached for ^177^Lu-FAP-2286 treatment groups after 42 days of monitoring (Fig. 6C). No ^177^Lu-FAP-2286–related body weight loss was observed during the study (Fig. 6B).

### Comparative imaging of FAP-2286 and FAPI-46

**Fig. 7:**
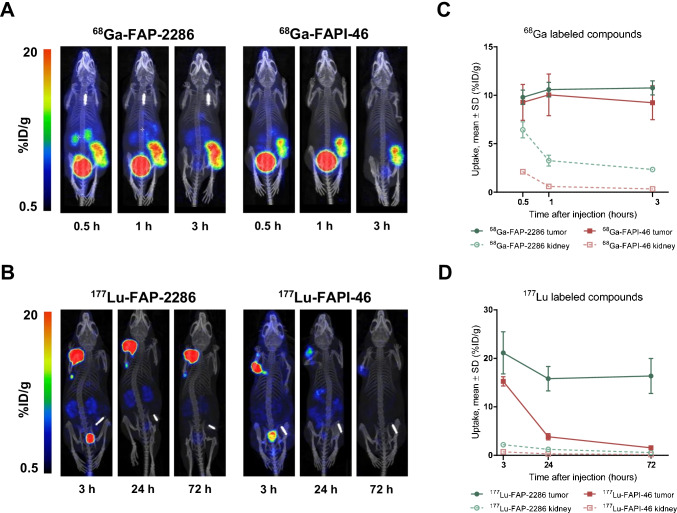
Biodistribution and tumor retention of FAP-2286 and FAPI-46 in HEK-FAP xenografts. Uptake of ^68^Ga-FAP-2286 and ^68^Ga-FAPI-46 (**A**) was assessed at 0.5, 1, and 3 h; and ^177^Lu-FAP-2286 and ^177^Lu-FAPI-46 (**B**) at 3, 24, and 72 h after dosing (*n* = 3 per group). One representative mouse at the 3 different timepoints is shown for FAP-2286 (left image) and FAPI-46 label radiotracers (right image). Time series plots show radiotracers accumulation as mean %ID/g ± SD at timepoints after injection in tumors and kidneys (**C**, **D**)

We compared the biodistribution of FAP-2286 against FAPI-46, the current lead therapeutic compound from the FAPI radiotracer series (28). ^68^Ga-FAP-2286 and ^68^Ga-FAPI-46 were rapidly enriched within the HEK-FAP xenografts with predominantly renal elimination (Fig. 7A, Supplementary Table S3). High tumor specificity was observed at all 3 timepoints assessed (0.5, 1, and 3 h p.i.). At 0.5 h after administration of ^68^Ga-FAP-2286 and ^68^Ga-FAPI-46, tumor uptake was 9.8 and 9.3 %ID/g, respectively. Accumulation of ^68^Ga-FAP-2286 and ^68^Ga-FAPI-46 was maintained at 3 h after injection with 10.8 and 9.2 %ID/g, respectively, and no significant difference in tumor distribution was observed between both compounds (Fig. 7C).

The ^177^Lu-radiolabeled FAP-2286 and FAPI-46 also demonstrated rapid accumulation within the HEK-FAP xenografts with low background signal at 3 h after dosing (Fig. 7B, Supplementary Table S3). However, significantly higher tumor retention of ^177^Lu-FAP-2286 compared to ^177^Lu-FAPI-46 was observed at 24 and 72 h p.i. (*P* < 0.001). Tumor uptake of ^177^Lu-FAP-2286 at the 3-h timepoint was 21.1 %ID/g and demonstrated durable retention with 16.4 %ID/g 72 h later. The time-integrated activity coefficient (TIAC) and absorbed dose were calculated to be 11.2 MBq^*^h/MBq and 2.8 Gy/MBq, respectively. In contrast, ^177^Lu-FAPI-46 accumulation decreased from 15.3 %ID/g at the 3-h timepoint to 1.6 %ID/g after 72 h resulting in a lower TIAC of 0.9 MBq*h/MBq and absorbed dose of 0.3 Gy/MBq (*P* < 0.001, Fig. 7D, Supplementary Table S3 and Fig. S4). Similar to the gallium-labeled compounds, normal tissues showed limited uptake and rapid clearance of the radiotracers with the highest level in the kidneys. A higher kidney accumulation was observed for ^177^Lu-FAP-2286 compared to ^177^Lu-FAPI-46 with the greatest difference at 3 h after injection with 2.2 %ID/g and 0.7 %ID/g, respectively, which decreased to 0.6 %ID/g and 0.2 %ID/g, respectively, at 72 h p.i. (Supplementary Table S3).

### Comparison of the efficacy of ^177^Lu-FAP-2286 and ^177^Lu-FAPI-46 in the HEK-FAP tumor model

**Fig. 8:**
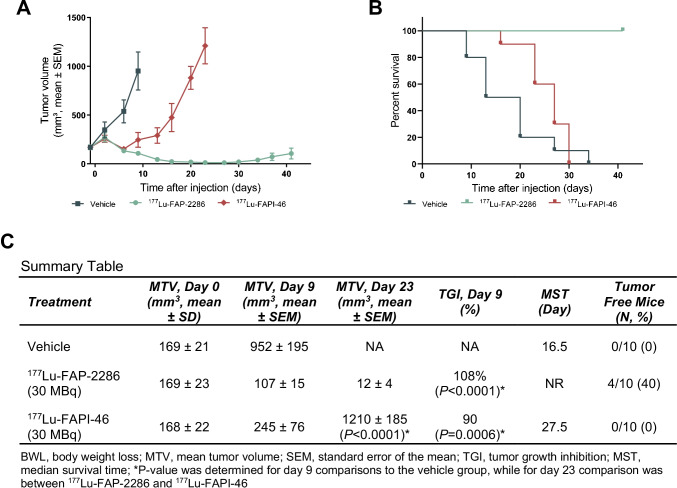
Efficacy of FAP-2286 and FAPI-46 in HEK-FAP xenografts. HEK-FAP tumor-bearing mice were treated with vehicle, ^177^Lu-FAP-2286, or ^177^Lu-FAPI-46 (30 MBq, *n* = 10 mice/group). Mean tumor volumes ± SEM (**A**) and survival curves (**B**) and a summary table (**C**) are shown

The efficacy of ^177^Lu-FAP-2286 and ^177^Lu-FAPI-46 was evaluated in the HEK-FAP model as a single 30 MBq dose administration. Tumors in the vehicle control group reached an MTV of 952 mm^3^ on day 9 p.i., the last timepoint when < 10% of the animals in the vehicle group had been sacrificed due to tumor volumes reaching humane endpoint criteria. In contrast, the MTV for ^177^Lu-FAP-2286 and ^177^Lu-FAPI-46 treatment groups were reduced to 107 and 245 mm^3^, respectively, on day 9 p.i. (Fig. 8A). Statistically significant antitumor activity was observed with TGI of 108% and 90% (*P* < 0.001) in mice treated with ^177^Lu-FAP-2286 and ^177^Lu-FAPI-46, respectively, relative to the vehicle control group.

HEK-FAP xenografts were monitored for regrowth out to the end of the study (day 41), when 4 mice out of 10 in the ^177^Lu-FAP-2286 treatment group had no measurable tumors (< 10 mm^3^), while all mice treated with ^177^Lu-FAPI-46 had tumors. In addition, there was a statistically significant difference in the MTV in animals treated with ^177^Lu-FAP-2286 (12 mm^3^, *n* = 10) versus ^177^Lu-FAPI-46 (1210 mm^3^, *n* = 9, *P* < 0.0001) on day 23 when < 10% of the animals in the treatment groups were sacrificed due to tumor volume (Fig. 8C). The MST for the ^177^Lu-FAP-2286 group was not reached while the MST for the vehicle control and ^177^Lu-FAPI-46 groups were 16.5 and 27.5 days, respectively (Fig. 8B).

### Cellular internalization and retention of FAP-2286 and FAPI-46

In order to gain more insight into the different tumor retention time of both molecules, AF488-labeled FAP-2286 and FAPI-46 were synthesized for studying cellular internalization, localization, and retention by live cell fluorescence microscopy. AF488 was conjugated to a free carboxy moiety of the DOTA for FAPI-46, and attached to the C-terminus of FAP-2286 since the chelator and metal complex may contribute to FAP-2286 binding affinity (Supplementary Methods).

**Fig. 9:**
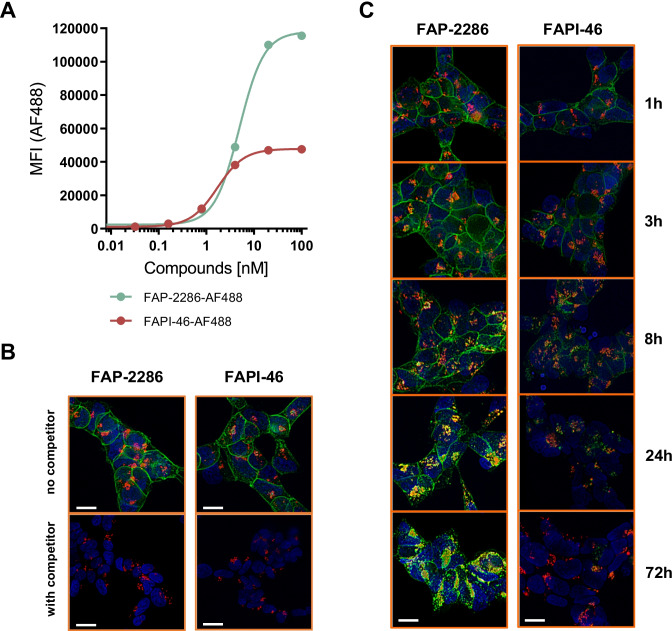
Cellular internalization and clearance of AF488-labeled FAP-2286 and FAPI-46 in HEK-FAP cells. HEK-FAP cells were incubated with various concentrations of AF488-labeled FAP-2286 or FAPI-46 for 1 h at 37 °C. MFI was measured by flow cytometry and plotted against compound concentration (**A**). HEK-FAP cells were incubated with 5 nM AF488-labeled FAP-2286 or FAPI-46 for 1 h at 37 °C (top images), and an excess of 5 µM unlabeled competitor compound was used for blocking (bottom images) (**B**). Cells were further incubated for an additional 1, 3, 8, 24, and 72 h (**C**). For visualization of lysosomal compartments, LysoTracker DeepRed was used. Cell nuclei were stained with Hoechst 33324. Images were taken with a Keyence fluorescence microscope. Scale bar 10 µm

Both compounds bound in a dose-dependent, saturable manner to HEK-FAP cells with half-maximal effective concentration (EC_50_) values of 4.9 nM for AF488-FAP-2286 and 1.7 nM for AF488-FAPI-46 (Fig. 9A). The binding of AF488-labeled compounds was completely blocked by increasing concentrations of unlabeled FAP-2286 or FAPI-46, suggesting FAP-specific binding and an overlapping binding site on FAP (Supplementary Fig. S5).

AF488-labeled FAP-2286 and FAPI-46 retention on FAP-expressing HEK293 cells was visualized by fluorescence microscopy. After 1-h incubation with 5 nM of AF488-FAP-2286 or AF488-FAPI-46 at 37 °C, compounds bound to the cell surface at comparable intensity indicated by a green staining of the cell membrane, which was blocked by competition with unlabeled ^nat^Lu-FAP-2286 or FAPI-46, respectively (Fig. 9B). Stained HEK-FAP cells were further incubated for additional 1, 3, 8, 24, and 72 h at 37 °C in compound-free medium (Fig. 9C). Intracellular structures with endosomal appearance were visible after 1 h of incubation indicated by green fluorescent puncta. From 3 h onward, the number of orange and yellow fluorescent endosomal structures increased indicating internalization into late endosomes or lysosomes and colocalization with LysoTracker. After 8 h of incubation, FAPI-46 had greater reduction in cell surface fluorescence and less intracellular fluorescence than FAP-2286. By 72 h, FAPI-46 was not detectable while FAP-2286 retained intracellular signal with little to no cell surface fluorescence.

## Discussion

FAP is an attractive cell surface target for radiopharmaceutical development given its tumor-restricted expression profile. While typically low to undetectable in most normal adult tissues, FAP is highly expressed on the cell surface in multiple cancer subtypes (6). In this study, the abundant expression of FAP as determined previously by others through mRNA and protein analyses was confirmed in tumor tissues of diverse origin, including pancreatic ductal adenocarcinoma, sarcoma, CUP, mesothelioma, cholangiocarcinoma, and breast cancer. While largely consistent across studies, some minor differences between FAP protein expression derived from TMA versus the larger tissue section immunohis to chemistry results were observed. For example, bladder cancer sections demonstrated stronger staining with median *H*-score of 25.0 (38% FAP positive, *n* = 26) than the TMA cores with 2.5 median *H*-score (*n* = 40). This discrepancy may be due to the metastatic bladder specimens assessed as whole sections (*n* = 21), whereas TMA bladder cores were from primary lesions; also, FAP expression in bladder metastasis has been reported to increase in immunotherapy-resistant tumors (35).

For most tumor specimens examined, FAP expression was restricted to the CAFs in the stromal cell compartment of the tumor. In comparison, in a large subset of sarcomas and mesotheliomas and smaller subset of epithelial derived cancers including esophageal, FAP expression was observed in tumor cells in addition to the CAFs population. These results are consistent with studies reporting tumors of mesenchymal origin express FAP in cancer and stromal cell populations (11,36). Furthermore, FAP expression as analyzed by immunohistochemistry directly correlated with data obtained by autoradiography of matched tumor specimens using ^111^In-FAP-2286 as the detection reagent, suggesting that FAP-2286 binds specifically to FAP.

Low-molecular-weight targeting approaches for radiotherapy, including small organic molecules and peptides, have the advantage of rapid tumor delivery and systemic renal clearance, augmenting efficacy and reducing the potential for myelosuppression. Of these compounds, the quinoline-based FAPI series of radiotracers have demonstrated compelling imaging results in patients (21–23,25). However, because of their short tumor residence time, FAPI radiotracers in their current form are not optimal for therapeutic applications when utilizing radionuclides with a half-life > 24 h. Currently validated small molecule radiotherapeutics such as LUTHERA® (^177^Lu-dotatate) and PSMA-617 employ ^177^Lu as a β-emitting radionuclide, which has a half-life of 6.7 days, to deliver a persisting radiation dose for therapeutic applications. Using the HEK-FAP mouse tumor model, we confirmed the lower tumor retention of FAPI-04 with a rapid clearance rate of approximately 50% every 24 h (Supplementary Fig. S6), consistent with fast washout kinetics reported previously (21,28,37).

To overcome the short retention time, second-generation molecules based on the same FAP-targeting moiety of UAMC1110 (31) employed in FAPI series of compounds have been developed including QCP02 (30), RPS-309 (38), and OncoFAP (29,39). While most are in preclinical testing, 2 radiotracers have been evaluated in patients as imaging agents: DOTA.SA.FAPi and FAPI‐46. With a squaramide linker for improving biological half-life, DOTA.SA.FAPi has demonstrated similar performance as ^18^F-FDG in diagnosing patients (40). Likewise, FAPI‐46, as a continuation on the FAPI series, showed improved tumor‐to‐organ ratios, resulting in enhanced image contrast (28). However, the increase in tumor retention time for FAPI-46 and DOTA.SA.FAPi was incremental, and further molecular modifications may be required to allow the optimal use of isotopes with long decay rates for therapeutic applications.

FAP-2286 was developed with the hypothesis that an improved tumor retention time could be achieved using a cyclic peptide to target FAP compared to small molecule–based radiotracers. FAP-2286 labeled with ^177^Lu displayed prolonged tumor retention (≥ 72 h) compared to FAPI-46 after injection in the HEK-FAP mouse model as measured by SPECT/CT image quantification, and yet maintained rapid system renal clearance as expected for small molecules. The longer tumor retention of FAP-2286 as compared to FAPI-46 resulted in 12- and 9-fold higher TIAC and absorbed dose delivered to the tumors, respectively, which ultimately lead to greater tumor inhibition.

Consistent with the SPECT imaging data, Alexa Fluor 488–derivatized FAP-2286 was retained in HEK-FAP cells and secluded in endosomes out to 72 h *in vitro*, whereas Alexa Fluor 488–derivatized FAPI-46 levels decreased over time starting at 8 h, despite both showing equivalent level of initial binding to HEK-FAP cells. A previous study reported similar results with a first-generation FAPI compound (FAPI-02) in HT1080-FAP cells (41). In binding and efflux experiments, ^177^Lu-FAPI-02 retained 34% of its initial radioactivity after 24 h of incubation, which was later demonstrated to be comparable to FAPI-04 (21).

The benefit of the long retention of FAP-2286 was observed not only in HEK-FAP but also in Sarc4809 PDX tumors after a single administration of ^177^Lu-FAP-2286. Compared to the vehicle control group, statistically significant antitumor activity evident by tumor shrinkage was observed from day 5 onward in the HEK-FAP mice of high- and low-dose treatment groups. Similarly, in the Sarc4809 PDX model of sarcoma, there was a statistically significant difference in the RTV between the high- and low-dose treatment versus control groups on day 19. The observed therapeutic effect in the treatment groups is attributed to the targeted delivery of the β-particle emitting radionuclide ^177^Lu to the tumor mass, since the nonradioactive ^nat^Lu-labeled compound had no effects. Tumor suppression is therefore presumed to be caused by radiation-induced DNA damage and subsequent apoptotic cell death of the FAP-expressing cells (42,43). In this scenario, the tumor model Sarc4809 would be representative of cancers of mesenchymal origin, where FAP is present on tumor cells contributing to their histologies and tumorigenesis (11) and ^177^Lu-FAP-2286 would render cytotoxic radiation to tumor cells.

Targeting FAP is not limited to tumor cells but can also be applied to the CAFs in the stroma that support tumor development and maintenance (6). In cancers of epithelial origin, FAP expression is mainly restricted to the CAFs in the tumor microenvironment, which possesses diverse functions, such as stromatogenesis, reciprocal signaling interactions with cancer cells, and crosstalk with tumor-infiltrating leucocytes (6,12). Because FAP-expressing CAFs play an essential role in tumorigenesis, their depletion has been associated with tumor shrinkage (44–46).

In addition to CAF-directed ablation by the β-particle emitting radionuclide payload, radiation-induced bystander effects and crossfire targeting of tumor cells can occur in the vicinity of CAFs, where the radiopharmaceutical accumulates (47). Tumor cells in the bordering areas adjacent to FAP-expressing CAFs are affected by radiobiological damage as a result of signals originating from irradiated cells (48), while the crossfire effect occurs within the range of the β-particles by traversing radiation penetrating several cell layers (49). Consequently, the radiotherapeutic approach using β-particles may be advantageous in terms of a broader destruction of the tumor mass over other FAP-targeting agents that only directly affect FAP-expressing CAFs. Further work using mouse tumor models with FAP-positive CAFs infiltration is warranted, and model development studies are on-going to recapitulate CAFs recruitment and maintenance in mice. However, due to the lower affinity of FAP-2286 towards murine FAP the utilization of a surrogate molecule with higher binding to murine FAP would be needed to enable translating the results to humans.

Apart from kidney uptake, which was related to the excretion of the radiolabeled peptide and rapidly cleared, limited accumulation of radioactivity in other non-target tissues was observed during imaging. This resulted in the high HEK-FAP tumor-to-background contrast in the ^177^Lu-FAP-2286 SPECT/CT images without treatment-related acute toxicity or body weight loss in the efficacy studies. However, the studies described presently were not designed to assess the long-term hematologic and renal toxicities or the secondary malignancies that have been reported in patients with targeted radiotherapies (50).

In the first-in-human experience of 11 patients, FAP-2286 has established its utility as a theranostic agent labeled with ^68^Ga for diagnosis and with ^177^Lu for treatment. PET/CT scans of ^68^Ga-FAP-2286 confirmed significant uptake in neoplastic lesions and low presence in normal tissues of patients with either pancreatic, breast, ovarian, or colorectal carcinomas. As a therapeutic radiopharmaceutical, ^177^Lu-FAP-2286 showed similar biodistribution by SPECT/CT as the imaging agent ^68^Ga-FAP-2286, and likewise, it had significant tumor uptake and long retention that appeared to improve symptoms manifested by pain reduction in 3 patients with advanced disease (51).

Taken altogether, FAP-2286 demonstrates compelling characteristics of a targeting agent with potent and selective FAP binding that leads to high tumor accumulation and substantial therapeutic efficacy. The preclinical studies as well as the first-in-human experience support further development of ^68^Ga-FAP-2286 and ^177^Lu-FAP-2286, and evaluation in patients with advanced solid cancers in the ongoing phase 1/2 clinical study LuMIERE (NCT04939610) for assessment of their safety, pharmacokinetics, dosimetry, and efficacy.

## Supplementary Information

Below is the link to the electronic supplementary material.

Supplementary file 1 (DOCX 1.28 MB)

## Data Availability

The data generated in this study are available within the article and its supplementary data files.
