# Supplementary material for: Preclinical evaluation of FAP-2286 for fibroblast activation protein targeted radionuclide imaging and therapy
Source: Eur J Nucl Med Mol Imaging. 2022 May 24;49(11):3651–67. doi: 10.1007/s00259-022-05842-5 (PMC9399058; doi:10.1007/s00259-022-05842-5)
Supplement: Supplementary file 1 — (DOCX 1.28 MB) [file 259_2022_5842_MOESM1_ESM.docx]

**Preclinical Evaluation of FAP-2286 for Fibroblast Activation Protein Targeted Radionuclide Imaging and Therapy**

Dirk Zboralski^1^, Aileen Hoehne^1^, Anne Bredenbeck^1^, Anne Schumann^1^, Minh Nguyen^2^, Eberhard Schneider^1^, Jan Ungewiss^1^, Matthias Paschke^1^, Christian Haase^1^, Jan L. von Hacht^1^, Tanya Kwan^2^, Kevin K. Lin^2^, Jan Lenore^2^, Thomas C. Harding^2^, Jim Xiao^2^, Andrew D. Simmons^2^, Ajay-Mohan Mohan^3^, Nicola Beindorff^3^, Ulrich Reineke^1^, Christiane Smerling^1^, and Frank Osterkamp^1^

^1^3B Pharmaceuticals GmbH, Berlin, Germany; ^2^Clovis Oncology, Inc., Boulder, Colorado, USA; and ^3^Berlin Experimental Radionuclide Imaging Center, Charité - Universitätsmedizin Berlin, Berlin, Germany

**Corresponding Author:** Dirk Zboralski, 3B Pharmaceuticals GmbH, Magnusstraße 11, D-12489 Berlin, Germany; Phone: +49-30-63924317; E-mail: dirk.zboralski@3b-pharma.com

# Supplementary Figures and Tables

**Supplementary Figure S1. FAP mRNA expression in human cancers.** FAP RNA expression in multiple tumor types by RNA-seq analysis is shown in dot plot. Median is represented by black horizontal line.

**
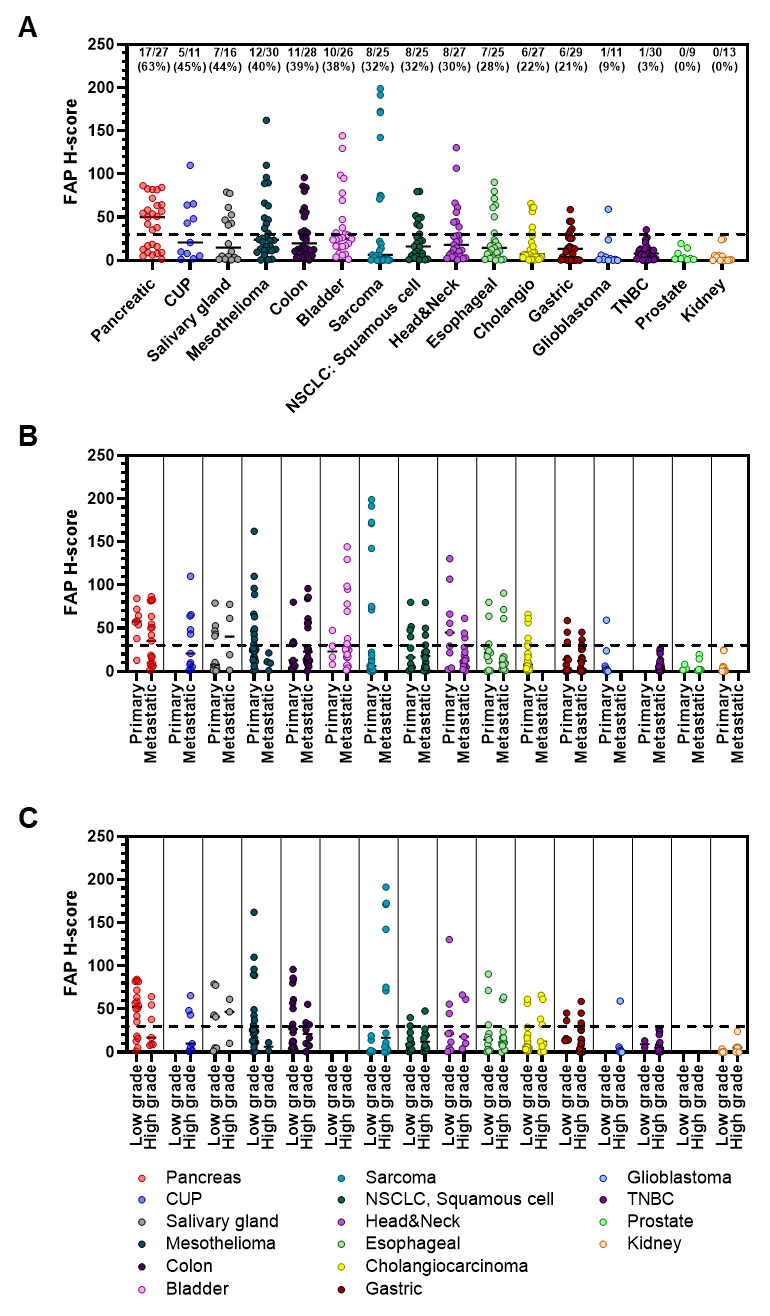
**

**Supplementary Figure S2. Quantification of FAP expression in whole tumor tissue sections.** Overall FAP staining (H-scores, Visiopharm); dot plots show the median score for each indication; horizontal line at H-score=30 shows cut-off for high FAP expression (A). Overall FAP staining by primary vs. metastatic biopsy site (B) and by low or high tumor grade (C); dot plots show the median score for each indication; horizontal line at H-score=30 shows cut-off for high FAP expression (H-scores, Visiopharm). CUP, cancer of unknown primary; NSCLC, non-small cell lung cancer; HNSCC, head and neck squamous cell carcinoma; TNBC, triple negative breast cancer

| *Inhibition of Human DPP4 and PREP Peptidase Activity* | | |
| --- | --- | --- |
| **Compound** | **Human DPP4 IC_50_ (nM)** | **Human PREP IC_50_ (nM)** |
| FAP-2286 | >10,000 | >1,000 |
| ^nat^Ga-FAP-2286 | >10,000 | >1,000 |
| ^nat^In-FAP-2286 | >10,000 | >1,000 |
| ^nat^Lu-FAP-2286 | >10,000 | >1,000 |
| IC_50_, half-maximal inhibitory concentration | | |

**Supplementary Table S1. *In vitro* characterization of FAP-2286 and its metal complexes against antitargets DPP4 and PREP.**

**
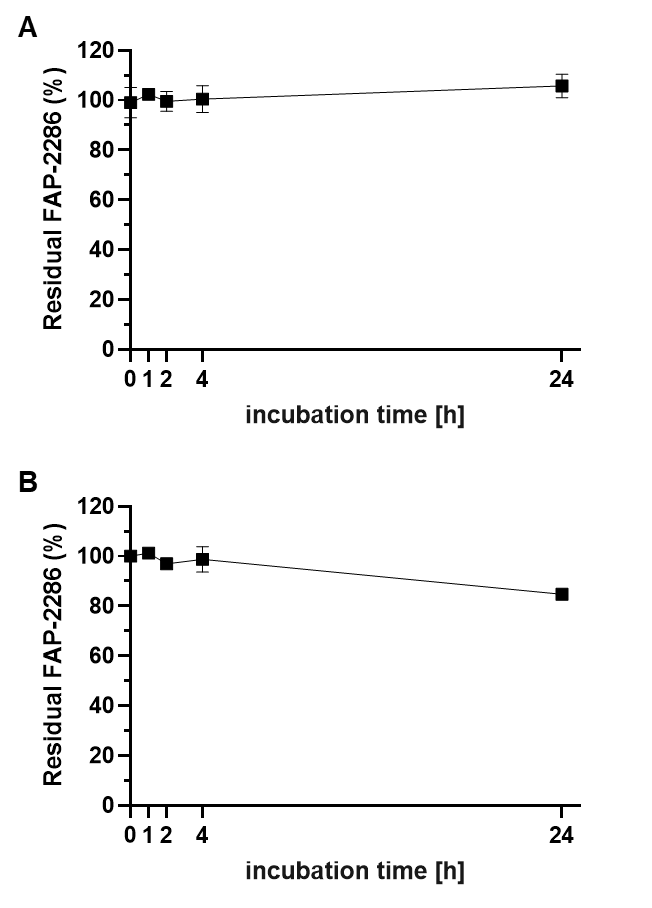
**

**Supplementary Figure S3. Stability of FAP-2286 in Plasma.** Residual FAP-2286 after incubation in human (A) and mouse (B) plasma.

| *^111^In-FAP-2286* | | | |  |
| --- | --- | --- | --- | --- |
| **Time p.i. (h)** | **Tumor %ID/g (mean ± SD)** | **Kidney %ID/g (mean ± SD)** | **T/K ratio (mean ± SD)** |  |
| 1 | 11.1 ± 4.3 | 3.9 ± 1.0 | 3.0 ± 1.3 |  |
| 3 | 11.6 ± 4.9 | 2.5 ± 0.4 | 4.6 ± 1.9 |  |
| 6 | 11.4 ± 6.6 | 2.2 ± 0.3 | 5.0 ± 2.8 |  |
| 24 | 9.7 ± 5.3 | 1.6 ± 0.2 | 5.9 ± 3.3 |  |
| 48 | 9.1 ± 5.0 | 1.3 ± 0.2 | 7.5 ± 4.8 |  |
| *^111^In-FAP-2286* | | | | |
| **Time p.i. (h)** | **Liver %ID/g (mean ± SD)** | **Shoulder %ID/g (mean ± SD)** | **Salivary Glands %ID/g (mean ± SD)** | **Blood Pool Surrogate %ID/g (mean ± SD)** |
| 1 | 0.3 ± 0.1 | 1.2 ± 0.6 | 0.6 ± 0.4 | 0.4 ± 0.1 |
| 3 | 0.2 ± 0.1 | 0.4 ± 0.1 | 0.2 ± 0.1 | 0.1 ± 0.1 |
| 6 | 0.1 ± 0.0 | 0.1 ± 0.1 | 0.2 ± 0.2 | 0.1 ± 0.0 |
| 24 | 0.1 ± 0.0 | 0.1 ± 0.0 | 0.0 ± 0.0 | 0.0 ± 0.0 |
| 48 | 0.1 ± 0.0 | 0.1 ± 0.0 | 0.0 ± 0.0 | 0.0 ± 0.0 |

**Supplementary Table S2. Uptake of ^111^In-FAP-2286 in the HEK-FAP mouse model.** Quantification of ^111^In-FAP-2286 uptake in HEK-FAP xenograft tumors (n=9) (A) and representative normal tissues, including kidney, liver, shoulder (non-tumor bearing), salivary gland, and blood pool surrogate (heart) (B).

**
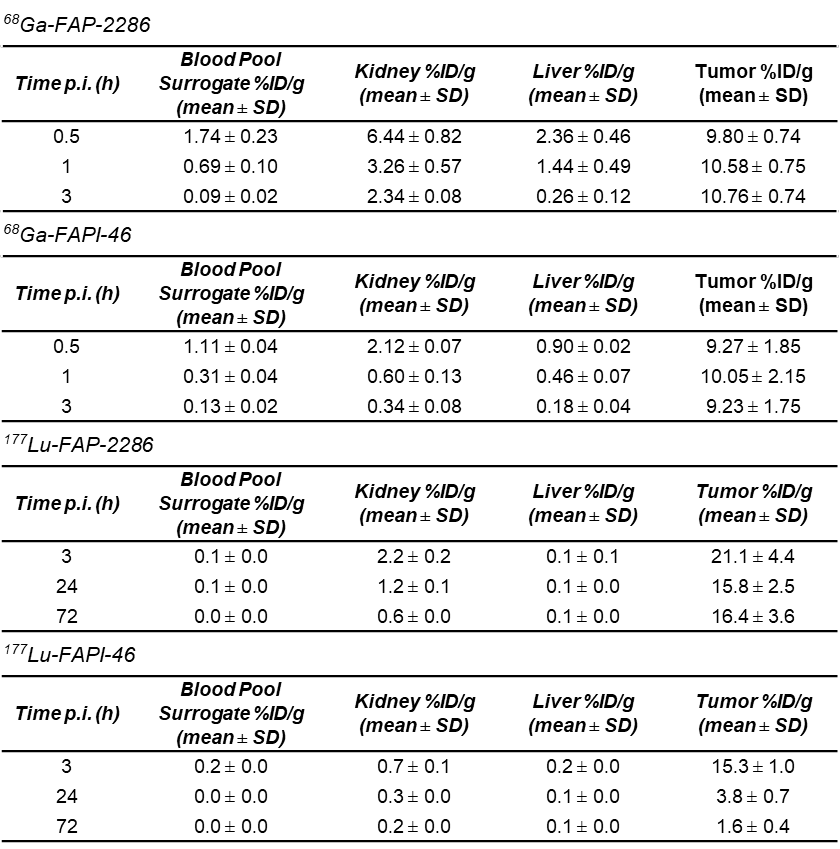
**

**Supplementary Table S3.** **Uptake of ^68^Ga and** **^177^Lu labeled FAP-2286 and FAPI-46 in the HEK-FAP mouse model.** Quantification of uptake in HEK-FAP xenograft tumors and normal tissues including kidney, liver and blood pool surrogate (heart) from the efficacy study shown in Fig. 4 (n=3).

**Supplementary Figure S4. HEK-FAP absorbed radiation doses.** (A) Mean HEK-FAP tumor uptake shown as fraction of injected dose (ID) for tracers ^177^Lu-FAP-2286 and ^177^Lu-FAPI-46 (n=3 per treatment group). Timepoints 3, 24, and -72 h post injection (p.i.) were determined by quantitative SPECT/CT; 72 h p.i. onwards was extrapolated by a one-phase decay fit (dashed lines). Tumor uptake curve fitting and self-dosimetry results are shown in table (B).

**
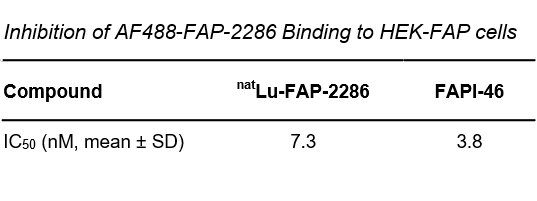

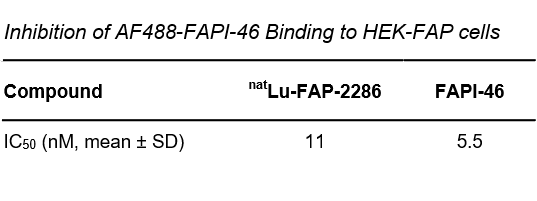
**

**Supplementary Figure S5. Binding inhibition of AF488-labeled FAP-2286 and FAPI-46 with unlabeled competitor compounds.** HEK-FAP cells were coincubated with 5 nM of either AF488-FAP-2286 or AF488-FAPI-46 with increasing concentrations of unlabeled competitor (^nat^Lu-FAP-2286 or FAPI-46) and analyzed by flow cytometry. MFI values are plotted against competitor concentrations for determination of the IC_50_ values.

**Supplementary Figure S6. Uptake of ^111^In-FAPI-04 in HEK-FAP xenograft model.** Quantification of ^111^In-FAPI-04 uptake in HEK-FAP xenograft tumors (n=6) and representative normal tissues, including kidney, liver, shoulder (non-tumor bearing), salivary gland, and blood pool surrogate (heart).

# Supplementary Materials and Methods

**Synthesis of FAP-2286**

The linear precursor sequence Hex-Cys-Pro-Pro-Thr-Gln-Phe-Cys-OH (**A,** Supplementary Figure M1) was synthesized by standard Fmoc-Solid-Phase peptide chemistry on a Trityl resin in a 50-µmol scale. After final resin cleavage and precipitation with chilled methyl-tert-butylether/cyclohexane (1/1) the crude peptide was lyophilized from water/acetonitrile.

Supplementary Figure M1: Cyclization and 2-aminoethanethiol introduction

The crude linear peptide **A** was dissolved in 10 mL of a 1:1 mixture of ethanol and acetonitrile. To this mixture, 35 µL of *N*,*N*-diisopropylethylamine and then 23.7 mg of 1,3,5-tris(bromomethyl)benzene (66.6 µmol, 1.3 equivalents [eq] compared to initial resin loading) were added. The solution was stirred for 1 hour, and then, 42.8 mg of 2-aminoethanethiol (555 µmol, 11 eq compared to initial resin loading) was added. After 1 hour, the solvents were removed by evaporation and the remainder dissolved in acetonitrile and water (20 mL, 1:1 mixture, containing 50 µL TFA). After lyophilization the crude product was purified by reversed phase high-performance liquid chromatography (RP-HPLC) to yield 17.8 mg (16.4 µmol) of the cyclic intermediate **B** (32.8%) as shown in Supplementary Figure M1.

Supplementary Figure M2: Final step of synthesis of FAP-2286: DOTA-coupling to intermediate **B**

To a solution of the intermediate **B** in 300 µL dimethyl sulfoxide (DMSO) 5 µL N,N-Diisopropylethylamine (DIPEA) were added to adjust the pH value to approximately 7.5–8. Then 19.0 mg of DOTA-NHS (hexafluorophosphate/trifluoroacetic acid salt - 25 µmol, 1.5 eq compared to the peptide intermediate) in 200 µL DMSO were added. During the course of the liquid chromatography/time-of-flight-mass spectrometry (LC/TOF-MS) monitored reaction 5 µL DIPEA was added 3 times to readjust the pH value to the starting value. After completion of the reaction the solution was subjected to RP‑HPLC purification to yield 20.44 mg (13.9 µmol) of FAP-2286 as shown in Supplementary Figure M2 (84.7% yield for the coupling of DOTA-NHS, 27.8% overall yield). Purity by UV-HPLC (230 nm): >95%, LC/TOF-MS: exact mass 1469.640 (calculated 1469.639), C_67_H_99_N_13_O_18_S_3_ (MW = 1470.780).

**Alexa Fluor 488 Labeling**

AF488-labeled ^nat^Lu-FAP-2286 was synthesized by 3B Pharmaceuticals and is a C-terminal elongated FAP-2286 derivative which is complexed with cold lutetium and comprises a C-terminal lysine. As last step of the synthesis the free C-terminal lysine was conjugated with AF488-dye utilizing AF488-tetrafluorophenyl ester.

AF488-labeled FAPI-46 was synthesized by AAT Bioquest by conjugation of a cadaverine-AF488 reagent to DOTA-carboxylic group of FAPI-46 (MedChemExpress) and final HPLC-purification to remove unreacted components along with bi- and tri-labeled compounds. The AF488-labeled FAPI-46 was obtained in low yield. Structurally it represents one AF488 dye-moiety linked via cadaverine to one of the FAPI-46-DOTA-carboxylic acid groups as amide bond.

**Immunohistochemistry**

The antibody SP325 (Abcam) was initially assessed for specificity by FAP staining patterns in healthy adult tissue (normal colon) and tumor samples (colon, lung, and breast cancers) as described by Hamos et al. (2014), Wikberg et al. (2013), Santos et al. (2009) and Tchou et al. (2013). A tissue slide containing both intrinsic positive and negative staining structures is included in every staining run. A matched isotype control antibody (Cell Signaling Technology, clone 3900S) serves as an extrinsic tissue negative control. For H-score assessment, the staining intensity was scored as 0, 1, 2, or 3 corresponding to the presence of negative, weak, intermediate, and strong brown staining, respectively. The average percentage positive was calculated for each intensity category and the following calculation was applied: H-score = (% of cells stained at intensity category 1 x 1) + (% of cells stained at intensity category 2 x 2) + (% of cells stained at intensity category 3 x 3). Image analysis scoring was previously validated against scoring performed by a pathologist.

**Autoradiography**

Autoradiographic evaluation was performed on 20 µm thick sections, attached to chrome-gelatin coated slides. ^111^In-labeled FAP-2286 was diluted in incubation buffer (0.17 M Tris, pH 8.2, 1% bovine serum albumin [BSA]) to the desired radioactivity concentration (2 × 10^5^ counts per minute [CPM]/mL), which was verified via gamma counting. In order to assess nonspecific binding, an adjacent slide was incubated in tracer solution admixed with 1 µM of unlabeled FAP-2286–related blocking peptide. After 2-hour incubation at room temperature, the slides were washed 4 times for 5 minutes in precooled wash buffer 1 (0.17 M Tris, pH 8.2, 0.25 % BSA) and 2 times in wash buffer 2 (0.17 M Tris, pH 8.2), before drying the sections for at least 30 minutes. Protein-specific binding was analyzed with a CCD camera system and the corresponding MCID analysis software (Interfocus, RRID:SCR_014278). Signal strength was measured once on the tissue with total tracer binding and once in the same region of interest on the sample for nonspecific binding. For the evaluation of every experiment, a separate standard curve (100–100,000 CPM) was recorded and tissue-bound radioactivity was calculated based on the resulting power equation using GraphPad Prism 8.4 (RRID:SCR_002798).

**Biacore assay**

Biacore CM5 sensor chips (GE Healthcare Life Sciences) were used for surface plasmon resonance (SPR) studies. Human FAP antibody (MAB3715, R&D systems) or mouse FAP antibody (MAB97271, R&D systems) was diluted in 10 mM acetate buffer, pH 4.5, to a final concentration of 50 µg/mL. Antibodies were directly immobilized onto the flow cells at 25°C using the Amine Coupling Kit Reagent solutions (GE Healthcare Life Sciences), according to the manufacturer’s recommendations. A preinstalled program for immobilization was used with an immobilization level of 7000 RU, flow rate of 10 µL/minute.

Recombinant human FAP (rhFAP; Sino Biological) and mouse FAP (rmFAP; R&D systems) were diluted in Running Buffer (150 mM NaCl, 10 mM HEPES, 0.005% TWEEN-20, pH 7.4; HBST) to a final concentration of 20 and 4 µg/mL, respectively. A five-fold dilution series of test compounds with concentrations ranging from 50 nM to 0.19 nM was injected over the sensor surface for 120 seconds at a flow rate of 30 µl/minute. The dissociation was monitored for 1800 seconds and the surface was regenerated with 10 mM glycine pH 2. SPR binding analyses for binary complexes were performed in single cycle kinetics (SCK) mode at 25°C. Following 3 SCK measurements, a baseline drift was assessed by injecting running buffer through a flow cell, with the antibody and FAP immobilized to the sensor surface.

For each test compound, SPR raw data in the form of resonance units (RU) were plotted as sensorgrams using the Biacore T200 control software (RRID:SCR_019718). The signal from the blank sensorgram was subtracted from that of the test compound sensorgram (blank corrected). The blank corrected sensorgram was corrected for baseline drift by subtracting the sensorgram of a SCK run without the test compound (running buffer only). The association rate (k_on_), dissociation rate (k_off_), dissociation constant (K_D_), and t_1/2_ were calculated from Blank-normalized SPR data using the 1:1 Langmuir binding model from the Biacore T200 evaluation software (GE Healthcare Life Sciences).

**Cell-based binding assay**

In order to determine binding of FAP-2286 and metal-chelates to FAP-expressing cells, a competitive fluorescence-activated cell sorting (FACS) binding assay was established. FAP-expressing human WI-38 fibroblasts (obtained from the European Collection of Authenticated Cell Cultures, ECACC, RRID:CVCL_0579) were cultured in Eagle’s Minimum Essential Medium (EMEM) including 15% fetal bovine serum, 2 mM L-Glutamine, and 1% nonessential amino acids and used within 20 passages. WI-38 cells were tested negative for mycoplasma contamination (Minerva Analytix GmbH) and were authenticated by ATCC cell line authentication service in July 2021. Cells were detached with Accutase (Biolegend, #BLD-423201) and washed in FACS buffer (PBS including 1% FBS). Cells were diluted in FACS buffer to a final concentration of 100,000 cells/mL and 200 µL of the cell suspension were transferred to a U-shaped bottom, polypropylene 96-well plate (Greiner). Cells were washed in ice-cold FACS buffer and incubated with 3 nM of FAP-2286-related C-terminally Cy5-labeled competitor peptide in the presence of various concentrations of FAP-2286 and its metal chelates at 4°C for 1 hour. Cells were washed twice with FACS buffer and resuspended in 200 µL FACS buffer. Cells were analyzed in an Attune NxT flow cytometer (Thermo Fisher Scientific). Median fluorescence intensities (Cy5 channel) were calculated by Attune NxT software (RRID:SCR_019590) and plotted against peptide concentrations. Four parameter logistic (4PL) curve fitting and IC_50_/pIC_50_ calculations were performed using ActivityBase software (IDBS, RRID:SCR_004077).

In order to determine binding of Alexa-Fluor488-labeled FAP-2286 and FAPI-46 to FAP-expressing cells, a fluorescence-activated cell sorting (FACS) binding assay was established. FAP-expressing HEK293 cells (InSCREENeX, RRID:CVCL_0045) were detached with Accutase (Biolegend, #BLD-423201) and washed in FACS buffer (PBS including 1% FBS). Cells were diluted in FACS buffer to a final concentration of 100,000 cells/mL and 200 µL of the cell suspension were transferred to a U-shaped bottom, polypropylene 96-well plate (Greiner). Cells were washed in FACS buffer and incubated with increasing concentrations of AF488-FAP-2286 or AF488-FAPI-46, respectively. In the competition assay, cells were incubated with 5 nM of AF488-labeled compounds in the presence of various concentrations of ^nat^Lu-FAP-2286 or FAPI-46 at 37°C for 1 hour. Cells were washed twice with FACS buffer and resuspended in 200 µL FACS buffer. Cells were analyzed in an Attune NxT flow cytometer (Thermo Fisher Scientific). Median fluorescence intensities (FITC channel) were calculated by Attune NxT software and plotted against peptide concentrations using GraphPad Prism 8.4.

**Protease activity assay**

Recombinant human FAP (R&D Systems, # 3715-SE) or mouse FAP (R&D Systems, # 8647-SE) was diluted in assay buffer (50 mM Tris, 1 M NaCl, 1 mg/mL BSA, pH 7.5) to a concentration of 3.6 nM. 25 µL of the FAP solution was mixed with 25 µL of a 3-fold serial dilution of the test compounds and incubated for 5 minutes in a white 96-well ProxiPlate (Perkin Elmer). As the specific FAP substrate, the FRET-peptide HiLyteFluor 488 - VS(D-)P SQG K(QXL 520) - NH2 was used (Eurogentec, Bainbridge, et al., Sci Rep, 2017, 7: 12524). 25 µL of a 30 µM substrate solution, diluted in assay buffer, was added. All solutions were equilibrated at 37°C prior to use. Substrate cleavage and increase in fluorescence (excitation at 485 nm and emission at 538 nm) was measured in a kinetic mode for 5 minutes at 37°C in a SpectraMax M5 plate reader (Molecular Devices). RFU/sec was calculated by SoftMax Pro software (RRID:SCR_014240) and plotted against peptide concentration. 4PL curve fitting and IC_50_/pIC_50_ calculations were performed using ActivityBase software (IDBS).

To test selectivity of FAP binding peptides toward both PREP and DPP4, protease activity assays were performed similarly to the FAP activity assay with following exceptions. PREP activity was measured with recombinant human PREP (R&D Systems, #4308-SE). As substrate 50 µM Z-GP-AMC (Bachem, # 4002518) was used. The DPP4 activity assay was performed in DPP assay buffer (25 mM Tris, pH 8.0), with recombinant human DPP4 (R&D Systems, #9168-SE). 20 µM of GP-AMC (Santa Cruz Biotechnology, #115035-46-6) was used as substrate. Fluorescence of AMC (excitation at 380 nm and emission at 460 nm) after cleavage was measured in a kinetic mode for 5 minutes at 37°C in a SpectraMax M5 plate reader (Molecular Devices).

**Plasma stability**

FAP-2286 was incubated in 50 µL of plasma with 3.8% sodium citrate as anticoagulant at a concentration of 10 µM. After 1, 2, 4, and 24 hours incubation at 37°C a suitable internal standard was added. FAP-2286 with plasma proteins were precipitated using 150 µL of a precipitation agent consisting of 77% 0.1 M zinc sulfate solution and 23% acetonitrile. Precipitation was carried out on ice for 30 minutes. After centrifugation at 18,200 relative centrifugal force (rcf) for 5 minutes, 100 µL of the supernatant was mixed with 10 µL of 1% formic acid and incubated for 5 minutes at 60°C to complete zinc complex formation for the free DOTA compound.

A nonincubated plasma sample spiked with 10 µM sample concentration was prepared in the same way and was used as the reference. A pure plasma sample was incubated for 24 hours as a blank sample. A further pure plasma sample was incubated for 24 hours, processed as described above and spiked directly before analysis to determine the recovery. The samples were analyzed using an Agilent 1290 UHPLC system coupled to an Agilent 6530 QTOF mass spectrometer.

Chromatographic separation was carried out on a Phenomenex Aeris Peptide XB-C18 stationary phase (50 × 2.1 mm, 1.7-µm particle size) using a linear gradient from 2% B to 41% B in 7 minutes with 0.1% formic acid as eluent A and acetonitrile as eluent B. Flow rate was 0.8 mL/min and the column was heated to 40°C. Mass spectrometry detection was performed in positive ion mode. Complete mass spectra were collected for the whole chromatographic in a range between m/z 100 and 300 with a sampling rate of 3 spectra/second.

For the quantitative data analysis, the Agilent Quantitative Analysis B.08.00 software (RRID:SCR_015040) was used. The ion currents of the doubly charged proton adducts of the compounds were extracted with a mass window of ±50 parts per million and the chromatographic peak was integrated. The concentration of the compound was determined by external matrix calibration using 6 calibration samples (1, 2.5, 5, 7.5, 10, and 12.5 µM). A quadratic regression was performed. Carryover was determined by analyzing the blank sample directly after the highest calibration sample and comparing the determined peak areas. Recovery was calculated as the ratio between the spiked concentration of the recovery sample and the concentration calculated from the peak area of this sample.

**^111^In-, ^177^Lu- and ^68^Ga-labeling**

15–150 MBq of ^111^InCl_3_ (in 0.02 M HCl) were mixed with 1 nmol of FAP-2286 or FAPI-04 (200 µM stock solution in 0.1 M HEPES pH 7 or 500 µM stock solution in water) per 15–30 MBq ^111^InCl_3_ and buffer (1 M sodium acetate/ascorbic acid buffer pH 5 or 1 M sodium acetate buffer pH 5, containing 25 mg/mL methionine for FAP-2286 labeling) at a final buffer concentration of 0.1 M. The mixture was shaken at 300 rpm, 80°C for 20–30 minutes. After cooling down, diethylenetriamine pentaacetate (DTPA) and polysorbate 20/TWEEN-20 were added at a final concentration of 0.2 mM and 0.1%, respectively.

0.3–1.2 GBq ^177^LuCl_3_ (in 0.04 M HCl) were mixed with buffer (1 M sodium acetate/ascorbic acid buffer pH 5, containing 25 mg/mL methionine for FAP-2286 labeling; 100 µL per GBq ^177^LuCl_3_). 1 nmol of FAP-2286 or FAPI-46 (500 µM stock solution in water) per 30–60 MBq ^177^LuCl_3_ was added. The mixture was shaken at 300 – 600 rpm, 90°C for 15–25 minutes. After cooling down, DTPA and TWEEN-20 were added at a final concentration of 0.2 mM and 0.1%, respectively. In some instances, purification by solid phase extraction utilizing a C18 SepPak column pre-conditioned with absolute ethanol (5 mL) and water (10 mL) was performed. The crude product was diluted with water to a total volume of 5 mL, trapped on the pre-conditioned column, washed with water (5 mL) and then eluted with 500 μL 50/50 ethanol/water (v/v). The final product was formulated with 0.9% sterile NaCl for injection containing 10 mg/mL ascorbic acid 7 to a final ethanol concentration of < 10%.

For ^68^Ga-labelings, the generator was eluted with 0.1 M HCl using a fractionated method. 750 μL ^68^Ga eluate (200-500 MBq; 0.1 M HCl) was mixed with 750 μL freshly prepared labeling buffer (1.0 M ammonium acetate / 0.125 M ascorbic acid 4:1, pH 4.0) and 400 μL 50 % EtOH and preheated. The activity was measured, and the amount of peptide was added to reach the desired molar activity (20 MBq/nmol). The mixture was shaken at 600 rpm, 95°C for 15 minutes. The purification column (Oasis HLB plus light cartridge column for FAP-2286 and a C18 SepPak light column for FAPI-46) was washed with 5 mL 99.9% ethanol followed by 10 mL water. Before purification, the reaction was diluted with 10 mL water and then added to the column. The column was washed with 2 mL water. The product was eluted from the column in 0.25 mL 99.9 % EtOH. The final product was formulated in 0.9% sterile NaCl for injection containing 10 mg/mL ascorbic acid, pH 7 to a final ethanol concentration of < 10%.

For assessment of labeling efficacy by TLC, an aliquot of the labeling solution was applied to a strip of iTLC-SG chromatography paper (Agilent) and developed in 0.1 M citric acid pH 5.4. The iTLC strip was then analyzed with a TLC scanner (Raytest). The radioactivity measured at the origin represents radiolabeled peptide, whereas the radioactivity at the solvent front represents free radionuclide. The radiochemical purity was analyzed by HPLC by injecting labeling solution onto either a Poroshell SB-C18 2.7 μm column (Agilent); eluent A: water, 0.1% TFA, eluent B: acetonitrile; gradient from 5% B to 70% B within 15 minutes, flow rate 0.5 mL/min; detector: NaI (Raytest), DAD 230 nm or a XBridge C18 3.5 μm, 4.6x50 mm column; eluent A: water, 0.1% TFA, eluent B: acetonitrile, 0.1% TFA; gradient from 5% B to 20% B within 1 minute, then from 20% B to 50% B within 7 minutes; flow rate 1.5 mL/min; detector: NaI (Raytest), DAD 220 nm. The peak eluting with the dead volume represents free radionuclide, the peak eluting with the peptide-specific retention time as determined with an unlabeled sample represents radiolabeled compound. Radiochemical purity was ≥90% at the end of synthesis.

**Animal housing and handling**

The animal studies were conducted in different laboratories. ^111^In-Imaging and efficacy studies using the HEK-FAP model and different doses of ^177^Lu-FAP-2286 as well as the Sarc4809 PDX efficacy study took place at Berlin Experimental Imaging Center (BERIC, Germany). The Sarc4809 model was originally derived from a primary lesion of undifferentiated pleomorphic sarcoma [1]. These studies were performed in either female swiss nude (Charles River Laboratories, France – ^111^In-Imaging), NMRI nude (Janvier Labs, France – PDX efficacy study) or SCID beige mice (Charles River Laboratories, Germany - ^111^In-Imaging). ^68^Ga-Imaging and comparative studies with ^177^Lu labeled FAP-2286 and FAPI-46 were performed at Minerva Imaging ApS, Denmark using female athymic nude mice (Charles River Laboratories, Germany). For all experiments animals were ordered with an age of approximately 6 weeks. Animals were allowed an acclimatization period of at least one week prior to inoculation. Animals were housed in an animal room with 12 hours light and 12 hours darkness with free access to food and water. A maximum number of 5 animals were housed in the same cage, cages were equipped with nesting material and plastic or paper houses for hiding. Each cage was labeled with study ID, group and animal numbers and test compound. For efficacy studies animals were randomized according to tumor size. Animals were checked daily for any signs of illness or abnormal behavior. Main humane endpoints were defined as tumor volumes > 1500 mm^3^, tumor burden > 10% of animal weight, body weight loss >15% and tumor ulceration. When reaching one of these criteria the animal was sacrificed by cervical dislocation. All animal experiments were approved either by the Regional Office for Health and Social Affairs Berlin (LAGeSo), Germany or the National Animal Experiments Inspectorate under the Ministry of Environment and Food of Denmark.

***In vivo* biodistribution**

Imaging and image analysis were performed as described. During the scan, mice were kept under isoflurane anesthesia, and body temperature was maintained under warm air. The following acquisition parameters were used for the respective imaging modality.

NanoSPECT/CT (Mediso), ^111^In-Imaging

| **Acquistion parameters SPECT** | |
| --- | --- |
| System | NanoSPECT/CT |
| Scan range | Whole body, 3-bed mouse hotel |
| Time per projection | 60 s |
| Aperture model, pinhole diameter | Aperture #2, 1.5 mm |
| **Reconstruction parameters SPECT** | |
| Method | HiSPECT (Scivis), iterative reconstruction |
| Smoothing | 35% |
| Iterations | 9 |
| Voxel size | 0.15 mm × 0.15 mm × 0.15 mm |
| **Acquisition parameters CT** | |
| System | NanoSPECT/CT |
| Scan range | Whole body, 3-bed holder (mouse hotel) |
| Scan duration | 7 minutes |
| Tube voltage | 45 kVp |
| Exposure time | 500 ms |
| Number of projections | 240 |

nanoScan PET/CT (Mediso), ^68^Ga-Imaging

| **Aquisition parameters PET** | |
| --- | --- |
| Animals per bed | 3 |
| Type of PET scanning | Static |
| Energy window (keV) | 400-600 |
| PET acquisition time (min) (15, 30 and 60 min) | 5 |
| PET acquisition time (min) (90 min) | 15 |
| PET acquisition start | 15, 30, 60 and 180 min after injection |
| Reconstruction | Tera-Tomo 3D with random, attenuation and scatter correction |
| **Aquisition parameters CT** | |
| Animals per bed | 3 |
| Type of scan | Helical |
| Projections | 480 |
| Pitch | 1 |
| X-ray power (kVp) | 50 |
| Exposure time (ms) | 300 |
| Reconstruction resolution (Voxel Size, Slice Thickness, Isotropic voxel size (µm)) | 250 |
| Binning | 1:4 |

NanoSPECT/CT (Mediso), ^177^Lu-Imaging

| **Aquisition parameters SPECT** | | | |  |
| --- | --- | --- | --- | --- |
| Animals per bed | 3 | | | |
| Type of SPECT scanning | Multi-pinhole | | | |
| Time per projection | 30s (3h), 60s (24h and 48h), 120s (72h) | | | |
| Energy windows (keV) | Peaks (keV) | | Full width (%) | |
|  | Primary | 208.4 | 20% | |
|  | Secondary | 112.9 | 20% | |
|  | Tertiary | 56.1 | 20% | |
| **Aquisition parameters CT** | | | |  |
| Animals per bed | 3 | | | |
| Type of scan | Helical | | | |
| Projections | 480 | | | |
| Pitch | 1 | | | |
| X-ray power (kVp) | 50 | | | |
| Exposure time (ms) | 300 | | | |
| Reconstruction resolution (Voxel Size, Slice Thickness, Isotropic voxel size (µm)) | 250 | | | |
| Binning | 1:4 | | | |

For quantification, VivoQuant (InviCRO) was used to draw regions of interest (ROIs) in relevant tissues, with a ROI drawn over the left ventricle of the heart to serve as blood pool surrogate. Percentage injected dose per gram of tissue (%ID/g) was calculated from the tissue-associated radioactivity determined in the ROI, the volume of the ROI, and the decay-corrected dose entered during preprocessing of the images in VivoQuant.

**Efficacy Studies.**

**Sarc4809 Patient-derived xenograft (PDX) efficacy study**. Female NMRI nu/nu mice (Janvier Labs) were subcutaneously implanted with Sarc4809 fragments (Passage 9), approximately 2x2x2 - 3x3x3 mm of size w/o Matrigel (Experimental Pharmacology & Oncology Berlin-Buch). Treatment started on day 22 post-transplantation.

**Calculation of tumor growth inhibition (TGI).** Percent TGI is defined as the difference between the mean tumor volume of the test group and the control group. TGI values are calculated as follows:

| TGIᵢ [%] = | (1 − | Tᵢ − T_0_ | ) × 100 |
| --- | --- | --- | --- |
|  |  | Vᵢ − V_0_ |  |

T*ᵢ* is the mean tumor volume of a treatment group on a given day *i*, T₀ is the mean tumor volume of the treatment group on the day treatment started (day 0), V*ᵢ* is the mean tumor volume of the vehicle control group on the same day as T*ᵢ*, and V₀ is the mean tumor volume of the vehicle group on the initial day of treatment (day 0).

**Calculation of relative tumor volume**. Relative tumor volume (RTV as %) is calculated according to the following formula: RTV = (T*ᵢ*/ T_0_) × 100, where T*ᵢ* is the tumor volume at day *i* and T_0_ is the tumor volume at day 0.

**Calculation of treatment to control value (T/C).** The treatment to control value (T/C in %) was calculated from the ratio of the mean RTV values of treatment versus control groups on day *x* multiplied by 100.

| T/C*ₓ* [%]  = | mean RTV*ₓ* treatment group | × 100 |
| --- | --- | --- |
|  | mean RTV*ₓ* control group |  |

**Calculation of median survival time.** The median survival time (MST) is the time at which the fractional survival equals 50%. MST was calculated using the GraphPad Prism software (Prism 8.4).

**Calculation of body weight loss.** Percent body weight loss (BWL) was calculated as follows:

| BWL*ᵢ* [%] = | (1 − | BW*ᵢ* | ) × 100 |
| --- | --- | --- | --- |
|  |  | BW_0_ |  |

BW*ᵢ* is the mean body weight of one group on a given day *i*, BW_0_ is the mean body weight of the same group on the day treatment started (day 0).

**Statistical analysis.** For the evaluation of the statistical significance of antitumor efficacy and body weight change in the HEK-FAP mouse model, the nonparametric Kruskal–Wallis test followed by Dunn’s method for multiple comparisons was performed using GraphPad Prism (Version 8.4). For comparison of MTV between low- and high-dose groups at day 42, the Mann–Whitney test was performed (GraphPad Prism 8.4).

For the evaluation of the statistical significance of antitumor efficacy (T/C) in the Sarc4809 PDX model based on RTV at day 19, the mixed effects model followed by Tukey’s multiple comparisons test was performed using GraphPad Prism (Prism 8.4). For comparison of RTV between low- and high-dose groups at day 42, the Mann–Whitney test was performed (GraphPad Prism 8.4).

For the evaluation of the statistical significance of FAPI-46 compared with FAP-2286 in the HEK-FAP mouse model, one-way ANOVA with Dunnett’s test for multiple comparisons was used to test treatment groups against the vehicle group and two-tailed unpaired t-test was used to compare the two treatment groups.

The log-rank (Mantel–Cox) test for survival curve comparison was used (GraphPad Prism 8.4). All *P* values <0.05 were considered statistically significant.

**Cellular internalization assay.** For flow cytometric assay, human FAP expressing HEK293 cells (HEK-FAP) were grown in a subconfluent monolayer and detached from cell culture flasks using Accutase. Cells were diluted to a final concentration of 4 x 10^5^ cells per mL. An aliquot of 200 µL of the cell suspension was transferred to a U-shaped non-binding 96-well plate, centrifuged at 500 g for 1 min at 4°C and washed with FACS buffer. For the EC_50_ assay, cells were incubated with increasing concentrations of AF488-labeled FAP-2286 or FAPI-46 for 1 h at 37°C. For the IC_50_ assay, cells were incubated with 5 nM of AF488-labeled FAP-2286 or FAPI-46 in the presence of increasing concentrations of ^nat^Lu-FAP-2286 or FAPI-46. Cells were analyzed in an Attune NxT flow cytometer. Median fluorescence intensities (FITC channel) were calculated using the Attune NxT software and plotted against test compound concentrations. Four parameter logistic (4PL) curve fitting as well as EC_50_ or IC_50_ calculations were performed using GraphPad Prism software.

For fluorescence microscopy assay, HEK-FAP cells were seeded in Poly-D-Lysine-coated 8-well chamber slides at 1 x 10^5^ cells per well and incubated for 3 days at 37°C, 5% CO_2_ in a humified incubator. Attached cells were incubated for 1 hour with 5 nM AF488-labeled FAP-2286 or FAPI-46 in incubation buffer at 37°C. For visualization of lysosomes and late endosomes, cells were stained with LysoTracker DeepRed (Thermo Fisher) for 30 minutes at 37 °C. In order to demonstrate specific binding and internalization, cells were co-incubated with 5 nM of AF488-labeled FAP-2286 or FAPI-46 and 5 µM of ^nat^Lu-FAP-2286 or FAPI-46.

Cells were washed three times and incubation buffer was added. Following various incubation periods, cells were washed with HBSS and incubated for 5 min with 1 µg/mL Hoechst 33342 dye for nuclei counterstaining at room temperature.

Fluorescence images were acquired by using a Keyence BZ‑X800E fluorescence microscope with 40x magnification.

- Image Size: 1920x1440
- Objective Lens: PlanApo_λ 40x 0.95/0.25-0.16mm :Default
- CH1 Exposure Time: 1/7.5s (Hoechst33342 channel)
- CH2 Exposure Time: 1/1.2s (AF488 channel)
- CH3 Exposure Time: 1/7.5s (LysoTracker Deep Red channel)
- Gain: +6dB
- Transmitted Light Power: 0
- Aperture Stop: 0%
- Mercury Quantity: 100%
- White Balance: OFF
- Contrast Correction: Black balance ON
- Filter: OFF
- Haze reduction: OFF
- Capturing Mode: Monochrome 12bit, normal capture
- Binning: OFF
- Number of Photos: 8
- Pitch: 0.8 µm

Images (8 per 0.8 µm pitch) were processed using Keyence BZ-X800 Analyzer software (RRID:SCR_017205) and following settings

- Full Focus: Standard Image
- Haze Reduction
  - Blur Size: 8
  - Brightness: 5.0
  - Reduction Rate: 0.90

**Tumor dosimetry**

Mean tumor time-integrated activity coefficients (TIAC) of three animals per treatment group, which underwent SPECT/CT image acquisition, were calculated. First, non-decay corrected tumor dose concentrations (in ID/g) were multiplied with the mean tumor mass (1 cm^3^= 1 g) which resulted in mean total tumor dose fractions (ID).

At timepoint zero (injection of ^177^Lu-tracer) no tumor uptake was assumed. To calculate the area under the curve from timepoint zero to infinity, the AUC from 0-72 h p.i. was computed by trapezoidal integration. The remaining activity was extrapolated beyond 72 h post injection by non-linear regression of the 24-72 h timepoints to infinity (one-phase decay fit in GraphPad Prism). The extrapolated values were also integrated and the sum of the AUC_0-72 h_ and AUC_72 h-infinity_ values resulted in the time-integrated activity coefficients (TIAC).

Next, tumor self-doses were calculated by using absorbed fractions for various sized spheres given in OLINDA 2.2. S-values were plotted against spheres with various masses ranging from 0.01 to 10 g. The data were fitted with a Power-Law function and variables a=80.31 and b=-0.9811 were determined in GraphPad Prism (power series $y=a*x^{b}).$ Finally, each TIAC was multiplied with the respective S-value to determine the tumor absorbed self-doses.

# Supplementary References

1. Hoffmann J, Schmidt-Peter P, Hänsch W, Naundorf H, Bunge A, Becker M, et al. Anticancer drug sensitivity and expression of multidrug resistance markers in early passage human sarcomas. Clin Cancer Res. 1999;5:2198-204.
